# Keratinization-related gene signature predicting survival and response to radiation in patients with HPV-negative head and neck squamous cell carcinoma via regulation of cornification and integrin signaling

**DOI:** 10.1186/s11658-025-00855-y

**Published:** 2026-01-29

**Authors:** Min Kyeong Lee, Harim Joo, Minji Bae, Yeonseo Lee, Joo-Kyung Noh, Young Chan Lee, Jung Woo Lee, Soonki Min, Moonkyoo Kong, Seong-Gyu Ko, Young-Gyu Eun

**Affiliations:** 1 Department of Biomedical Science and Technology, Graduate School, Kyung Hee University, Seoul, Republic of Korea; 2 Department of Otolaryngology-Head and Neck Surgery, Kyung Hee University School of Medicine, Kyung Hee University Medical Center, #23 Kyungheedae-Ro, Dongdaemun-Gu, Seoul, 02447 Republic of Korea; 3 Department of Oral and Maxillofacial Surgery, School of Dentistry, Kyung Hee University, Seoul, Korea; 4 Department of Radiation Oncology, Kyung Hee University School of Medicine Kyung Hee University Medical Center, Seoul, Republic of Korea; 5 Department of Preventive Medicine, College of Korean Medicine, Kyung Hee University, Seoul, Republic of Korea

**Keywords:** HPV-negative HNSCC, KRGS, Radiation therapy, Keratinization

## Abstract

Head and neck squamous cell carcinoma (HNSCC) is a highly heterogeneous malignancy associated with poor prognostic outcomes. Despite ongoing efforts to identify reliable biomarkers for prognosis, the clinical utility of these markers remains limited owing to the need for further validation and deeper mechanistic insights. In this study, we developed and validated a novel keratinization-related gene signature (KRGS) to predict prognosis and radiation therapy (RT) response in human papillomavirus (HPV)-negative HNSCC using data from The Cancer Genome Atlas (TCGA). The 16-gene KRGS effectively stratified patients with HNSCC into two subgroups with significantly differing survival outcomes. KRGS^low^, characterized by low KRGS expression, exhibited poorer survival and reduced sensitivity to RT, while KRGS^high^, with high KRGS expression, was associated with more favorable survival outcomes and enhanced RT responsiveness. Functional validation, both in vitro and in vivo, demonstrated that keratinization activation through all-*trans* retinoic acid (ATRA) treatment upregulated the cornified envelope and sensitized HNSCC cells to RT. The enhanced response to RT was further associated with the upregulation of eight KRGS-related genes and increased expression of involucrin (IVL), a key regulator of terminal differentiation during cornification. Notably, the combination of ATRA and IR reduced radioresistance in HNSCC cells, which was linked to the downregulation of integrin alpha-1 (ITGA1) expression. These findings provide new insights into the role of keratinization in modulating radioresistance and suggest that KRGS-driven activation of keratinization, in combination with RT, may represent a promising therapeutic strategy to overcome resistance in HNSCC.

**Supplementary Information:**

The online version contains supplementary material available at 10.1186/s11658-025-00855-y.

## Introduction

Head and neck cancer (HNC) is the sixth most commonly diagnosed cancer worldwide, with approximately 630,000 new cases and over 350,000 deaths annually [1]. More than 90% of HNCs are classified as head and neck squamous cell carcinoma (HNSCC), which arises from the mucosa of the oral cavity, oropharynx, hypopharynx, and larynx [2]. The primary risk factors for HNSCC include tobacco use and alcohol consumption, which together account for nearly 80% of cases globally [3]. In addition, human papillomavirus (HPV) plays a significant role in the tumorigenesis of HNSCC [4]. HPV-associated HNSCC is now recognized as a distinct entity from HPV-negative HNSCC owing to differences in epidemiology, risk factors, treatment strategies, and prognosis [5]. The latest edition of the American Joint Committee on Cancer (AJCC) staging system for HNSCC reflects this distinction by incorporating HPV status into its classification [6]. Patients with HPV-positive HNSCC generally have better prognosis, exhibiting lower locoregional recurrence rates and higher overall survival (OS) compared with those with HPV-negative HNSCC. Furthermore, HPV-positive HNSCC is more responsive to chemotherapy and radiation therapy (RT), leading to a survival rate exceeding 80%, whereas the 5-year survival rate for HPV-negative HNSCC remains below 50% [7, 8]. The poor prognosis of HPV-negative HNSCC is attributed to its heterogeneity, which contributes to variability in clinical outcomes such as OS, recurrence, and resistance to treatment [9]. In recent years, considerable efforts have been made to establish a robust classification system for HPV-negative HNSCC, aiming to define clinically and molecularly homogeneous subgroups [9, 10]. However, significant challenges remain in translating these molecular subtypes into clinical practice. Furthermore, despite advancements in treatment, current therapies still lack tumor specificity, often causing collateral damage to critical structures in the head and neck region. This leads to substantial morbidity, even among long-term survivors [11, 12]. Given these challenges, there is an urgent need for targeted therapeutic strategies specifically designed for HPV-negative HNSCC. Therefore, this study aims to address the unique complexities of HPV-negative HNSCC and explore novel approaches to improve patient outcomes.

Keratinization begins with the proliferation of basal cells in the basal layer of the epithelium [13]. As these cells migrate toward the surface, they undergo progressive maturation and differentiation, accumulating keratin proteins while losing their nuclei [14]. Eventually, they form a cornified layer composed of keratinized, anucleate cells, which can be detected histologically as keratin pearls or dyskeratotic regions in squamous cell carcinoma [15]. In HNSCC, the presence of keratinization may influence tumor behavior, prognosis, and response to therapy. Some studies suggest that malignant cells may undergo hyperkeratinization, which could negatively affect treatment outcomes [16, 17]. Conversely, the degree of keratin differentiation may also enhance treatment efficacy in certain cases [18]. Additionally, keratinization has been implicated in reducing the likelihood of metastasis to regional lymph nodes [19]. Several studies have explored the relationship between keratinization and cancer prognosis, primarily focusing on histological characteristics and patient survival [20, 21].

However, the molecular signatures associated with keratinization and their impact on patient outcomes remain poorly understood. Identifying molecular subtypes linked to keratinization could enable better therapeutic stratification and more accurate predictions of prognosis and treatment response. To address this need, this study developed molecular signatures associated with keratinization in HPV-negative HNSCC using data from The Cancer Genome Atlas (TCGA). The study identified clinically distinct subtypes of HPV-negative HNSCC on the basis of keratinization-related gene expression patterns. Furthermore, key regulatory mechanisms associated with keratinization were uncovered, providing insights into potential therapeutic strategies to overcome RT resistance in HPV-negative HNSCC.

## Materials and methods

### Data acquisition

Gene expression and clinical data from TCGA (*n* = 451) were downloaded from the UCSC Cancer Genomics Browser (https://xena.ucsc.edu/public). Additional datasets were obtained from the National Center for Biotechnology Information Gene Expression Omnibus (GEO) database (http://www.ncbi.nlm.nih.gov/geo), including the Leipzig cohort (GSE65858, *n* = 270; Institute for Medical Informatics, Statistics, and Epidemiology) [22], the MDACC cohort (GSE42743, *n* = 74; MD Anderson Cancer Center), and the FHCRC cohort (GSE41613, *n* = 97; Fred Hutchinson Cancer Research Center) [23]. The Kyung Hee University (KHU) cohort (*n* = 47) was obtained from the Department of Otolaryngology-Head and Neck Surgery, School of Medicine, Kyung Hee University. All experiments were conducted with the written informed consent of each subject. This study was approved by and conducted in accordance with the policies of the Institutional of Kyung Hee University Medical Center. Additionally, the study methodologies were approved by the Institutional Review Board (IRB) of Kyung Hee University Medical Center (IRB: 2018–05–046–12) and adhered to the principles outlined in the Declaration of Helsinki. The gene expression data of HNSCC cell lines from the Cancer Cell Line Encyclopedia (CCLE) dataset were obtained from the DepMap Portal (Broad Institute, Cambridge, MA, USA) [24]. The pathological and clinical characteristics of patients in these four cohorts are summarized in Supplementary Table S1.

### Analysis of genes related to poor prognosis in HNSCC and identification of keratinization-related gene signature

First, to identify genes related to poor prognosis in HNSCC, we utilized gene expression data from HPV-negative patients in the TCGA database. Using each gene as a variable, a Cox proportional hazards (CoxPH) model was applied to determine which genes affected survival rates. Statistically significant genes (*p* < 0.05) were selected, resulting in 3950 genes. Next, to identify genes associated with keratinization, we used the Gene Ontology (GO) database (GO: 00310216). We identified 16 genes that overlapped between these two analyses. To characterize the keratinization-related subtypes, clustering analysis was performed using the ConsensusClusterPlus package [25]. The expression data of the 16 keratinization related gene signature (KRGS) were used as the basis for determining the molecular subtypes of the samples. A hierarchical clustering algorithm with 1 – Pearson correlation was employed as the metric distance, and 1000 bootstraps were conducted in the training set to ensure the robustness and reliability of the clusters. A grid search was performed, with the number of clusters varying from two to five, to determine the optimal clustering solution. The optimal number of clusters was selected on the basis of the evaluation of the cumulative distribution function (CDF) and consensus matrix, which reflect the stability and consistency of the clusters across bootstraps. The CDF plot and consensus matrix were examined to identify the most stable cluster solution. Subsequently, patients with HNSCC were divided into two subgroups: KRGS^low^ and KRGS^high^. To compare the survival differences between the two subgroups, Kaplan–Meier survival analysis was performed using the “survival” and “survminer” R packages. Kaplan–Meier curves were used to generate *p*-values and hazard ratios (HR) with 95% confidence intervals (CI) through log-rank tests.

### Validation of keratinization-related gene signature in independent cohorts

The gene expression signature was validated by classifying patients with HNSCC from independent cohorts using the Bayesian compound covariate predictor (BCCP) algorithm [26]. Gene expression data from the TCGA cohort (training set) were applied to the BCCP algorithm to generate discriminators. The performance of the BCCP algorithm was assessed using cross-validation methods, including leave-one-out cross-validation. Validation of the gene signature was subsequently conducted in the independent FHCRC, Leipzig, MDACC, and KHU cohorts. Predictive model construction and validation were carried out using the BRB-ArrayTools (https://brb.nci.nih.gov/BRB-ArrayTools/) [27].

### Cox proportional hazards regression analysis and construction of the nomogram

To evaluate whether the risk score could serve as an independent prognostic indicator for patients with HNSCC, univariate and multivariate CoxPH analyses were performed. This analysis included 451 patients with HPV-negative HNSCC from the TCGA cohort with available clinicopathological data. Univariate and multivariate CoxPH models were used to identify independent prognostic factors associated with survival of patients with HNSCC. The results of the CoxPH are reported as HRs, 95% CIs, and *p*-values. A nomogram was then constructed to visualize the results of the multivariate CoxPH analysis. Variables with a *p*-value < 0.05 on multivariate analysis were included in the nomogram, which was developed using the “rms” package in R to predict 3-year and 5-year OS rates. The predictive performance of the nomogram was assessed using bootstrapped calibration curves and quantified by the *C*-index. The *B* and *m* parameters were set to 1000 and 30, respectively, for model validation.

### Gene set enrichment analysis

Gene set enrichment analysis (GSEA) was conducted to explore potential pathways associated with the prognosis of HNSCC based on the 16 genes of the KRGS. The analysis was performed using the GSEA software (https://www.gsea-msigdb.org/gsea/index.jsp) with the human ENSEMBL-Gene-MsigDB chip as the chip platform [28]. Pathways were considered significantly enriched if the false discovery rate (FDR) was less than 0.05 following 1000 random permutations. This cutoff criterion was applied to identify key biological pathways associated with the gene signature and its prognostic relevance in HNSCC.

### Cell lines and cell culture

SNU1066 (cat. no. 01066), SNU46 (cat. no. 00046), and YD8 (cat. no. 60501) cell lines were obtained from the Korea Cell Line Bank (KCLB, Seoul, Republic of Korea). The HSC2 cell line (cat. no. CVCL_1287) was obtained from the Japanese Collection of Research Bioresources (JCRB, Osaka, Japan). SCC9 and CAL27 cell lines were purchased from the American Type Culture Collection (ATCC). Cells were passed every 3 days and maintained in culture for up to ten passages or a maximum of 4 weeks. Mycoplasma removal was performed using Mycoplasma Removal Agent (MP Biomedicals, Santa Ana, CA, USA). All cell lines were cultured at 37 °C in an atmosphere of 5% CO_2_. All cells were purchased within the last 5 years, and their identities were confirmed by short tandem repeat (STR) profiling by KCLB. SNU1066, SNU46, YD8, and HSC2 cells were cultured in Roswell Park Memorial Institute (RPMI1640, Corning, Manassas, VA, USA), SCC9 cells were cultured in Dulbecco’s modified Eagle medium (DMEM)/F12 (Corning). CAL27 and radioresistant CAL27 (CAL27-RR) cells were cultured in DMEM-high glucose. The media were supplemented with 10% fetal bovine serum (FBS, Corning) and 1% penicillin–streptomycin (PS, Corning).

### Development of radioresistant HNC cell lines and RNA-sequencing

The radioresistant cell line was established from the parental cell line by subjecting it to weekly exposures of 2 Gy ionizing radiation (IR) over a period of 15 weeks, accumulating a total radiation dose of 80 Gy. The development of radioresistance was confirmed through colony-forming assays (CFA) to assess the cell line’s ability to survive and proliferate after radiation exposure. RNA sequencing of radioresistant cell lines was performed. Total RNA was extracted from the cell lines using TRIzol^®^ reagent (Invitrogen, Carlsbad, CA, USA), following the manufacturer’s protocol. Complementary DNA (cDNA) was synthesized from the extracted RNA using the Tetro cDNA Synthesis Kit (Takara Bio Inc., Shiga, Japan). For transcriptomic analysis, high-quality RNA samples with an RNA integrity number (RIN) > 7.0 were selected for library construction. Library preparation was performed using 1 μg of total RNA per sample with the Illumina TruSeq^®^ Stranded mRNA Sample Prep Kit (Illumina Inc., San Diego, CA, USA, #RS-122–2101). The libraries were quantified using the KAPA Library Quantification Kit for Illumina platforms (Kapa Biosystems, Wilmington, MA, USA, #KK4854), following the qPCR Quantification Protocol Guide, and their quality was assessed using the TapeStation D1000 ScreenTape system (Agilent Technologies, Palo Alto, CA, USA, #5067–5582). Indexed libraries were subsequently sequenced on the Illumina NovaSeq platform (Illumina Inc.), generating paired-end (2 × 100 bp) reads. Sequencing, preprocessing, and transcriptome analysis were performed by Macrogen Inc. (Seoul, Korea).

### MTT assay

To evaluate the impact of all-*trans* retinoic acid (ATRA) on cell viability, 3-(4,5-dimethylthiazol-2-yl)−2,5-diphenyltetrazolium bromide (MTT) assays were conducted using the EZ-Cytox cell viability assay kit (Daeli Lab Service, Seoul, Republic of Korea). The HNSCC cells were seeded at a density of 5000 cells per well in 96-well plates and then treated with ATRA (1, 10, 5, 20, 50, 100, and μM/L). Following a 24-h incubation period in a humidified atmosphere containing 5% CO_2_ at 37 °C, cell viability was assessed using the MTT assay, with absorbance measured at 570 nm. ATRA was obtained from Sigma-Aldrich (CAS.no 302–79-4, Burlington, MA USA).

### Colony formation assay

CFA was performed to evaluate the cells’ ability to form colonies. Cells were seeded in 6-well plates at a density of 300 cells per well in growth medium supplemented with 10% FBS and 1% PS, and incubated at 37 °C. After 24 h, the cells were irradiated with doses of 0, 2, 4, or 8 Gy. Following irradiation, cells were cultured at 37 °C for 10–14 days. Subsequently, colonies were stained with Crystal Violet for 10 min at room temperature. All experiments were performed in triplicate. The statistical significance of differences between dose responses was determined using a two-way analysis of variance (ANOVA).

### Western blot

HNSCC cells were lysed in radioimmunoprecipitation assay (RIPA) buffer (50 mM Tris/HCl, 150 mM NaCl, 2 mM ethylenediaminetetraacetic acid (EDTA), and 1% Triton™ X-100), supplemented with protease inhibitors (Roche, Mannheim, Germany) and phosphatase inhibitors (Sigma-Aldrich). Protein concentrations were determined using a bicinchoninic acid (BCA) protein assay kit (Thermo Fisher Scientific, Waltham, MA, USA). For Western blotting, 10–50 μg of protein was mixed with sodium dodecyl sulfate (SDS) sample buffer (Invitrogen), boiled for 10 min at 100 °C, and electrophoresed on an 8–15% gradient bis–Tris gel. Subsequently, the proteins were transferred to a polyvinylidene fluoride (PVDF) membrane (Millipore, Billerica, MA, USA). The membrane was blocked with Tris-buffered saline (TBS) containing 5% nonfat dry milk, followed by overnight incubation at 4 °C with the following primary antibodies: anti-mixed lineage kinase domain-like protein (MLKL, 1:1000; Cell Signaling Technology, Danvers, MA), anti-cysteine-aspartic protease 3 (caspase 3, 1:1000; Cell Signaling Technology), anti-myeloid cell leukemia 1 (MCL1) (1:1000; Cell Signaling Technology), anti-antigen kiel 67 (Ki-67, 1:1000; Cell Signaling Technology), anti-involucrin (IVL, 1:1000; Abcam, Cambridge, CB4, UK), and β-actin (1:1000; Santa Cruz Biotechnology, Santa Cruz, CA, USA). After washing, the membrane was incubated with a species-specific horseradish peroxidase-conjugated secondary antibody (1:3000; Cell Signaling Technology) for 1 h at room temperature. The membrane was then rinsed three times with Tris-buffered saline with Tween 20 (TBS-T), and enhanced chemiluminescence (ECL) substrate (Amersham Cytiva, USA) was applied. Protein bands were detected using a ChemiDoc imaging system (Cytiva, USA). Quantification of Western blot data was performed using ImageJ software (National Institutes of Health, Bethesda, MD, USA).

### Quantitative real-time PCR

For the assessment of mRNA expression, quantitative real-time polymerase chain reaction (qRT-PCR) was performed. Total RNA was isolated using TRIzol following the manufacturer’s instructions (GeneAll, Seoul, Republic of Korea), and real-time qRT-PCR was conducted using an Applied Biosystems StepOne real-time PCR system. The PCR amplification conditions were as follows: 40 cycles of 95 °C for 15 s and 65 °C for 1 min, followed by thermal denaturation. The expression levels of each gene were normalized to GAPDH mRNA and quantified using the 2^−ΔΔCt^ method. Primer sequences used in the study are provided in Supplementary Table S2. Each sample was analyzed in triplicate.

### Cornified envelope assay

Cells were cultured in standard medium (control) or treated with ATRA. Following treatment, cells were harvested using trypsinization, washed with serum-containing medium, and resuspended in serum-free medium. The total number of cells in the suspension was determined using a hemocytometer. After 48 h, cells were washed twice with PBS to remove calcium. To induce the formation of cornified envelopes, cells were treated with 5 µM MgCl_2_ and 10 µM CaCl_2_. Subsequently, cells were incubated at 37 °C for 2 h with 10 µM calcium ionophore ionomycin (Sigma-Aldrich) to facilitate the formation of the cornified envelope. Following ionophore treatment, cells were collected by centrifugation and resuspended in PBS containing 1% SDS and 10 mM DTT. The cell suspension was then incubated at 90 °C for 15 min. The number of cornified envelopes was quantified under a microscope.

### In vivo studies

Female BALB/c nude mice, each weighing 20 ± 2 g, were purchased from JunbioTech (Daegu, Korea) and housed in a temperature- and humidity-controlled facility with a 12-h light/dark cycle, and mice were provided with food and water ad libitum. The study was conducted in accordance with the guidelines of the Kyung Hee Medical Center Institutional Animal Care and Use Committee (KHMC-IACUC-23–003-02). A total of 30 mice were used in this study and randomly assigned to six groups, with 5 mice in each group. CAL27-P or CAL27-RR cells (1.0 × 10^6^ cells in 0.1 ml saline) were subcutaneously injected into the right thighs of the mice, and tumors were allowed to grow. After 3 days, treatment began with either PBS (1 mL) or ATRA (2 μM/L), administered intraperitoneally for five consecutive days each week. In addition, tumors were exposed to fractionated IR (2 Gy daily for 5 days, totaling 10 Gy). This process mimicking the typical radiation protocol used for patients over the course of a week. The dosage of ATRA administered to mice was determined on the basis of prior studies, using levels shown to be nontoxic in mice [29]. The mice’s body weight and tumor size were recorded twice a week, and tumor growth was monitored by measuring the longest and shortest diameters at right angles using electronic calipers. Tumor volumes were then calculated using the following formula: formula volume = [(short)^2^ × (long)]/2.

### Immunohistochemistry staining

Mouse tumor samples were embedded in paraffin and sectioned into 4-μm-thick slices for immunohistochemistry (IHC) analysis. Antigen retrieval was performed by treating the sections with 3% hydrogen peroxide, followed by incubation with 0.25% pepsin. The sections were then incubated with a blocking solution containing 5% bovine serum albumin (BSA) for 30 min. After blocking, the sections were incubated overnight at 4 °C with a primary antibody against IVL (1:100 dilution) in 5% BSA solution. Following primary antibody incubation, sections were washed three times with PBS-T and then incubated with a polymer-horseradish peroxidase conjugated secondary antibody. Antibody binding was visualized using a 3,3′-diaminobenzidine (DAB) substrate-chromogen system. The stained sections were examined under an Olympus IX71 inverted microscope. For quantitative analysis, IHC staining was assessed using ImageJ software. The total area of the tissue was measured by adjusting the hue and saturation values to their maximum, while the brightness was set to select all tissues. To quantify the IHC-stained area, the color values were adjusted to identify only the stained regions, and the stained area was measured. The percentage of the IHC-stained area was calculated by dividing the IHC-stained area by the total area and multiplying by 100.

### Single-cell RNA-seq analysis

Single-cell RNA sequencing (scRNA-seq) data were obtained from the GSE140042 dataset, comprising 42,400 cells derived from seven patients with HNSCC, including both primary tumor and lymph node metastasis specimens. The Seurat package in R was used for data processing and quality control [30]. Primary cell clusters were identified using the FindNeighbors and FindClusters functions with a resolution parameter set to 0.5. To investigate the KRGS-defined subgroups at the single-cell level, we performed bulk RNA-seq-informed module scoring. First, the average expression of a predefined 16-gene KRGS signature was calculated for each bulk RNA-seq sample. On the basis of receiver operating characteristic (ROC) curve analysis, the optimal cutoff for distinguishing high- and low-expression groups was determined using the Youden index. This classification was then applied to single-cell RNA-seq data using the AddModuleScore function in Seurat (version 4.3), which computes a module score for each cell by aggregating the expression of the input gene set and comparing it with randomly selected control gene sets. Cells were stratified into “high” and “low” KRGS activity groups on the basis of quantile thresholds of the module scores. The distribution of KRGS expression across cell types in KRGS^low^ and KRGS^high^ was visualized using uniform manifold approximation and projection (UMAP) and violin plots [31].

### Estimation of immune-cell type fractions

Cell-type identification by estimating relative subsets of RNA transcripts (CIBERSORT) is a deconvolution algorithm that infers the cellular composition of complex tissues on the basis of normalized gene expression profiles. In this study, we utilized CIBERSORT (https://cibersort.stanford.edu/) in conjunction with the LM22 leukocyte signature matrix to quantify the proportions of immune cell types between KRGS^low^ and KRGS^high^ of the KRGS in HNSCC samples from the TCGA dataset. Normalized gene expression data were analyzed using the CIBERSORT algorithm with 1000 permutations. The statistical significance of the deconvolution results was determined using a *p*-value threshold of < 0.01. Among the 22 immune cell types defined in the LM22 matrix, 19 cell types with detectable expression in the dataset were included for the final analysis.

### Statistical analysis

The Kaplan–Meier method was used to generate OS, disease-specific survival (DSS), and recurrence-free survival (RFS) curves for each subgroup within each cohort. The log-rank test was applied to compare OS, DSS, and RFS rates between the subgroups. In the in vitro analysis, numerical data are presented as the mean ± standard deviation (SD) from at least three independent experiments. For comparisons between two experimental groups, a two-tailed unpaired Student’s *t*-test was applied. To compare three or more experimental groups, ANOVA with appropriate post hoc tests was used. A *p*-value of less than 0.05 was considered statistically significant. Statistical analysis and chart creation were performed using R version 4.2.1 and Prism 10 (GraphPad, CA, USA).

## Results

### Identification and validation of a keratinization-related gene signature in HPV-negative HNSCC

**Fig. 1:**
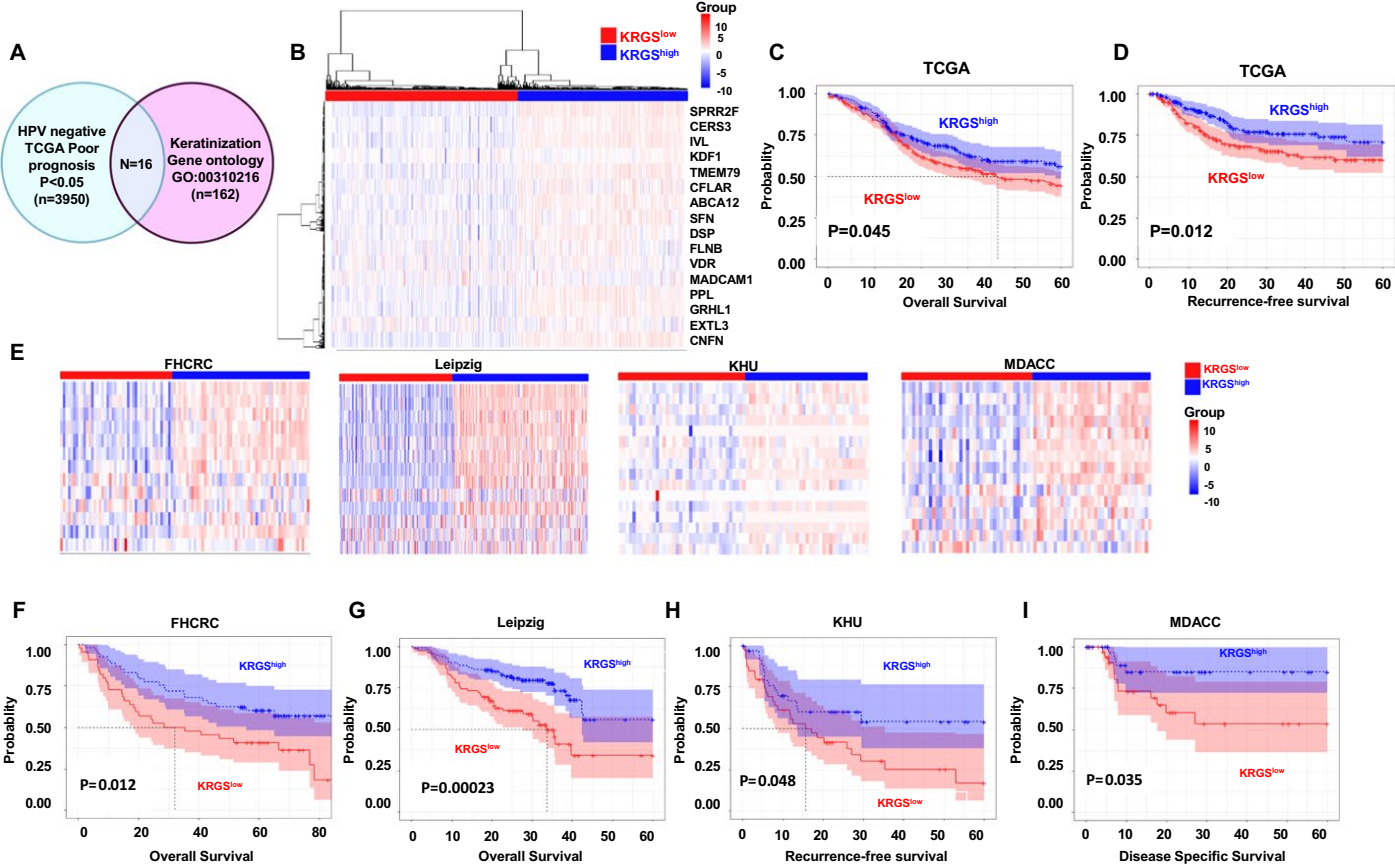
Performance of the KRGS in predicting OS in the TCGA discovery and validation cohorts. **A** Venn diagram analysis identified overlapping representative genes of the KRGS derived from the keratinization GO category and survival-associated genes determined through CoxPH. **B** Heatmap illustrating the expression patterns of 16 KRGS across the two subgroups in TCGA. Red indicates high expression levels, while blue represents low expression levels. Kaplan–Meier plots depicting prognostic differences in **C** OS and **D** RFS between the two subgroups in the TCGA cohort. Statistical significance was assessed using the log-rank test. **E** Heatmap illustrating the expression patterns of KRGS across the two subgroups in the validation cohorts. Kaplan–Meier curves showing prognostic differences in OS in the FHCRC (**F**) and Leipzig (**G**) cohorts, RFS in the KHU (**H**) cohort, and DSS in the (**I**) cohort between the two subgroups. Statistical significance was assessed using the log-rank test

To identify genes associated with patient survival, we applied the CoxPH model to the HPV-negative HNSCC dataset from TCGA, identifying 3950 genes with *p*-values < 0.05. We then examined genes related to keratinization using the GO database (GO:00310216) and identified 16 overlapping genes between the keratinization GO category and the genes identified by the Cox model (Fig. 1A). These 16 keratinization-related genes were collectively defined as the KRGS. We then performed consensus clustering on 451 patients with HPV-negative HNSCC in the TCGA cohort on the basis of KRGS expression levels. The consensus CDF plot of the consensus matrices revealed two distinct molecular subtypes (KRGS^low^ versus KRGS^high^, Fig. 1B). The CDF curve exhibited a clear plateau at *K* = 2, indicating that this was the optimal number of subgroups. (Supplementary Fig. 1 A, B). Moreover, selecting *K* = 2 minimized the ambiguity between subtypes (Supplementary Fig. 1 C). A significant difference in OS was observed between the two subgroups: patients in KRGS^low^ had worse outcomes than those in KRGS^high^ (OS: 48.5% versus 65.3% at 5 years, *p* = 0.045, Fig. 1C). Similarly, KRGS^low^ exhibited a significantly lower RFS rate compared with KRGS^high^ (RFS: 61.9% versus 81.3% at 5 years, *p* = 0.012, Fig. 1D).

These findings were further validated in multiple independent cohorts (FHCRC, Leipzig, KHU, and MDACC), using a BCCP predictor implemented in BRB-ArrayTools. The validation results were consistent with the initial findings from the TCGA cohort, and the heatmaps of KRGS expression across the four cohorts closely resembled those observed in the TCGA cohort (Fig. 1E). Moreover, KRGS^low^ patients exhibited significantly shorter OS than those in the KRGS^high^ group in both the FHCRC and Leipzig cohorts (FHCRC: OS: 44.6% versus 72.7% at 5 years, *p* = 0.012; Leipzig: OS 20.5% versus 57.3% at 5 years, *p* = 0.00023, respectively; Fig. 1F, G). Similarly, KRGS^low^ demonstrated significantly worse RFS in the KHU cohort compared with KRGS^high^ (RFS: 33.4% versus 87.8% at 5 years, *p* = 0.048; Fig. 1H) and poorer DSS in the MDACC cohort (DSS: 54.0% versus 88.3% at 5 years, *p* = 0.035; Fig. 1I). These findings underscore the robustness and predictive power of KRGS in prognosticating outcomes for patients with HPV-negative HNSCC.

### Independent prognostic and radiation therapy response-predictive value of the keratinization-related gene signature in HPV-negative HNSCC

**Fig. 2:**
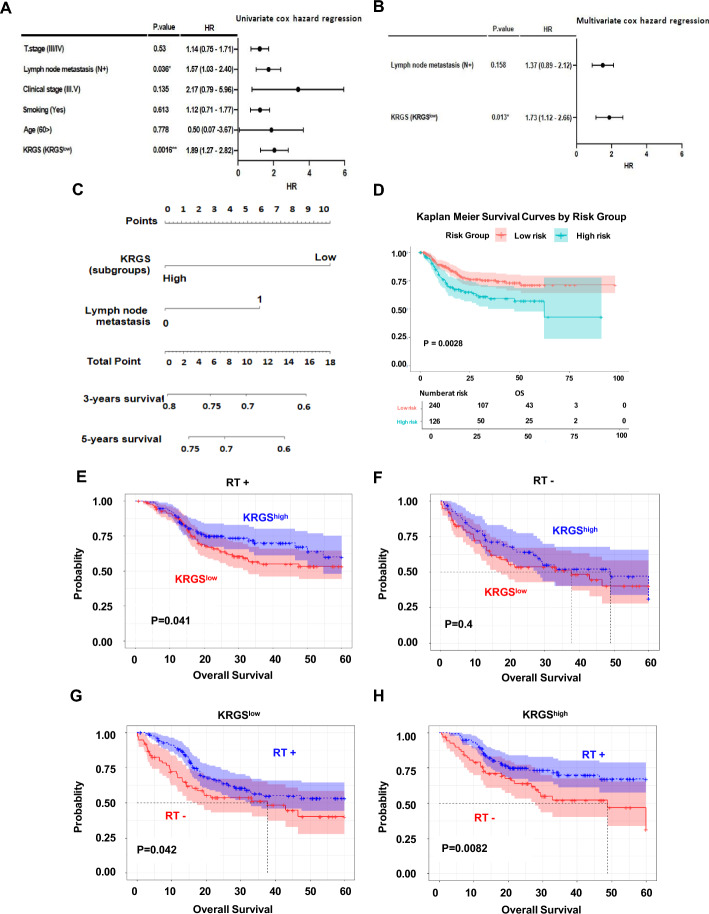
Prognostic value of KRGS and its association with radiation therapy response in HPV-negative HNSCC. **A** Forest plot showing the hazard ratios of KRGS along with clinicopathological variables in the univariate CoxPH analysis. **B** Multivariate CoxPH analysis illustrating the HR of KRGS in conjunction with clinicopathological variables. **C** Nomogram predicting survival in patients with HNSCC. The model was developed to estimate 3- and 5-year overall survival on the basis of KRGS and lymph node metastasis as prognostic factors. **D** Kaplan–Meier plots depicting 5-year OS in high- versus low-risk patients, with statistical significance assessed using the log-rank test. **E** Kaplan–Meier plot comparing OS between KRGS^low^ and KRGS^high^ patients who received RT. **F** Kaplan–Meier plot comparing OS between KRGS^low^ and KRGS^high^ patients who did not receive RT. **G** Kaplan–Meier plot comparing OS between patients who received or did not receive RT within KRGS^low^. **H** Kaplan–Meier plot comparing OS between patients who received or did not receive RT within KRGS^high^. Statistical significance was assessed using the log-rank test

To assess the prognostic impact of KRGS in the context of clinicopathological features, univariate and multivariate CoxPH analyses were performed in the HPV-negative TCGA cohort. Univariate analysis identified the KRGS subgroup (KRGS^low^ versus KRGS^high^) and lymph node metastasis (N− versus N+) as significant predictors of survival (Fig. 2A). Subsequent multivariate analysis, incorporating only variables that were statistically significant in the univariate model, revealed that KRGS was the only independent prognostic factor for OS in patients with HPV-negative HNSCC (Fig. 2B). Taken together, our results validate the robustness of KRGS as a prognostic marker, demonstrating its superior predictive power compared with conventional clinicopathological features. These findings highlight KRGS as a clinically relevant and independent predictor of survival in HPV-negative HNSCC.

To further enhance the predictive accuracy of KRGS, we assessed its performance in combination with other significant clinicopathological factors. We developed a clinically applicable risk nomogram, incorporating KRGS and lymph node metastasis, to estimate survival probability in patients with HPV-negative HNSCC. A higher total score in the nomogram, on the basis of the assigned risk points for each factor, was associated with worse 3-year and 5-year OS rates. Patients with lymph node metastasis and KRGS^high^ were assigned 6 points, whereas patients classified as KRGS^low^ of KRGS were assigned 10 points. (Fig. 2C) To evaluate the discriminatory power of this nomogram, we performed Kaplan–Meier survival analysis, stratifying patients on the basis of median risk scores derived from the nomogram (KRGS + lymph node metastasis). Patients in the high-risk group exhibited significantly poorer 5-year OS rates compared with those in the low-risk group (OS: 43.8% versus 73.9% at 5 years, *p* = 0.0028 Fig. 2D). To validate the predictive accuracy of the model, we assessed its performance using discrimination indices and calibration plots for 3-year and 5-year survival. The calibration plots demonstrated excellent agreement between observed outcomes and predicted survival probabilities (Supplementary Fig. 2 A, B). These results further reinforce the clinical significance of KRGS, demonstrating its superior predictive power in determining survival outcomes for patients with HPV-negative HNSCC.

We next investigated whether the KRGS subgroups predict response to RT. The HPV-negative TCGA cohort included patients who received RT as part of their standard care. The cohort was divided into two groups: patients who received RT (*n* = 248) and those who did not (*n* = 144). We found that KRGS^low^ showed significantly worse OS compared with KRGS^high^ in patients who received RT (OS: 44.0% versus 64.4% at 5 years, *p* = 0.041, Fig. 2E). However, KRGS^low^ and KRGS^high^ were not associated with survival differences in patients who did not receive RT. (OS: 44.0% versus 64.4% at 5 years, *p* = 0.4, Fig. 2F) To further assess the association between KRGS and RT, we performed an interaction test for OS, which revealed a significant interaction between KRGS subgroup and RT (*p* = 0.003431). Notably, a survival benefit from RT was observed in both KRGS^low^ and KRGS^high^, respectively (KRGS^low^, OS: 33.7% versus 65.5% at 5 years, *p* = 0.042, Fig. 2G; KRGS^high^, OS: 33.7% versus 65.5% at 5 years, *p* = 0.0082, Fig. 2H). These findings suggest that KRGS subgroups may serve as predictive biomarkers for RT response in HPV-negative HNSCC.

### Association of KRGS with tumor stage in HPV-negative HNSCC

We next sought to validate KRGS by comparing the early and advanced tumor stage groups. The HPV-negative TCGA cohort was dichotomized into early tumor stage (stage I/II, *n* = 103) and advanced tumor stage (stage III/IV, *n* = 328). KRGS was compared between patients in the early tumor stage and advanced tumor stage groups. We found that KRGS^low^ patients in the advanced tumor stage showed significantly worse RFS compared with KRGS^high^ (RFS: 48.9% versus 71.3% at 5 years, *p* = 0.03, Supplementary Fig. 3 A) However, there was no significant difference of RFS between KRGS^low^ and KRGS^high^ in the early tumor stage. (RFS: 52.9% versus 90.0% at 5 years, *p* = 0.27, Supplementary Fig. 3B). These results suggest that KRGS is associated with advanced tumor stage.

### Association of keratinization and integrin-mediated signaling pathways in KRGS of HPV-negative HNSCC

To evaluate the molecular pathways and mechanisms associated with KRGS, we performed GSEA. Not surprisingly, the top negatively enriched mechanism in KRGS^low^ was keratinization (NES = −1.6768, *p* = 0.0005, Supplementary Fig. 4 A). We next examined the rank-scored mechanisms in GSEA. The top five negatively enriched mechanisms in KRGS^low^ included keratinization, epidermis development, skin development, keratinocyte differentiation, and epithelium development (Supplementary Fig. 4B). Eight downregulated genes in KRGS^low^ compared with KRGS^high^ were identified (*CNFN*, *SPRR2F*, *PPL*, *ABCA12*, *SFN*, *IVL*, *CERS3*, and *TMEM79*, Supplementary Fig. 4 C). These results suggest that the downregulated keratinization-related genes in KRGS^low^ may be associated with poor prognosis. Next, we confirmed the positively enriched mechanisms in KRGS^low^. The top positively enriched mechanism in KRGS^low^ was the integrin-mediated signaling pathway (Supplementary Fig. 4D). Five major positively enriched mechanisms included integrin-mediated signaling pathway, regulation of response to external stimulus, positive regulation of signaling, regulation of immune system process, and receptor clustering (Supplementary Fig. 4E). Together, these mechanisms highlight the potential molecular basis through which KRGS may influence prognosis in HPV-negative HNSCC.

### Modulating keratinization to overcome radiation resistance in HNSCC cells

**Fig. 3:**
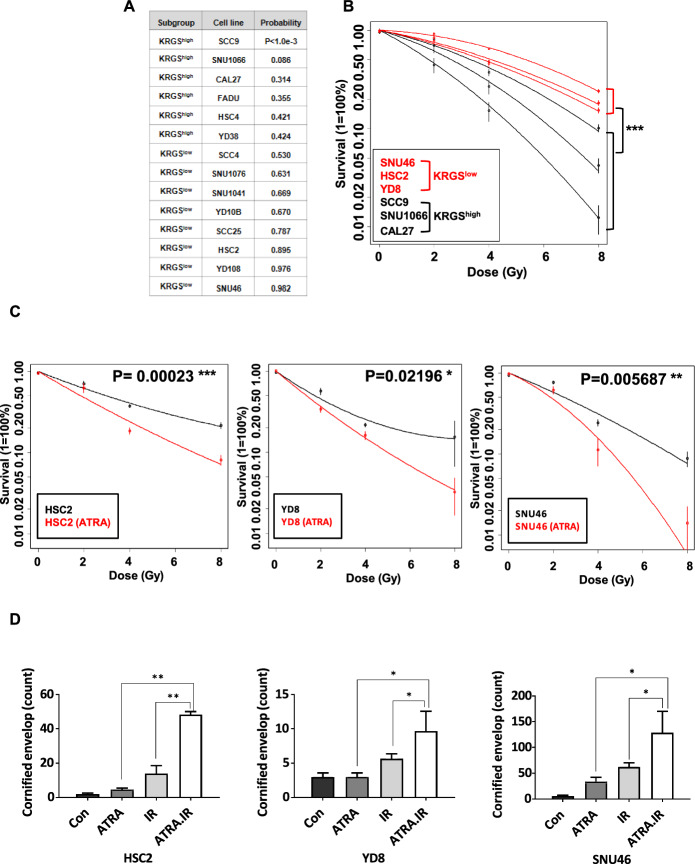
Enhanced radiation sensitivity by keratinization activation in HNSCC cell lines. **A** Classification of HNSCC cell lines on the basis of KRGS (*n* = 14 in the CCLE dataset). HNSCC cell lines were stratified according to the BCCP predictor. **B** CFA demonstrating the relative sensitivity differences among six HNSCC cell lines (HSC2, SNU46, YD8, CAL27, SNU1066, and SCC9) to IR over a range of 0 to 8 Gy, as depicted in the log–linear plot. The experiments were performed at least three times. Statistical significance was determined by ANOVA. Plating efficiency was higher than 12%. **C** CFA demonstrating increased radiation sensitivity in ATRA-treated KRGS.^low^ cell lines (HSC2, SNU46, and YD8) to IR over a range of 0 to 8 Gy, as depicted in the log-linear plot. Each point represents the means of three replicates. Statistical significance was determined by ANOVA. Plating efficiency was higher than 12%. **D** Cornified envelope assay in control, ATRA-treated, IR-irradiated, and ATRA with IR-treated cells. Cornified envelopes were counted under a microscope. The *x*-axis represents the treatment groups: Con (untreated control), ATRA (ATRA treatment), IR (irradiation), and ATRA.IR (combined ATRA and irradiation treatment). The *y*-axis indicates the number of cornified envelopes. Error bars represent the SD. Statistically significant were determined unpaired Student’s *t*-test. *p < 0.05; **p < 0.01; ***p < 0.001

Because established cancer cell lines are the most practical experimental models, we applied KRGS to the gene expression data of HPV-negative HNSCC cell lines from the CCLE dataset. The 14 cell lines from the CCLE dataset, suitable for laboratory application, were stratified into two subgroups on the basis of KRGS. Eight cell lines were classified into KRGS^low^, and six cell lines into KRGS^high^ (Fig. 3A). We selected three cell lines from each subgroup and conducted further in vitro functional studies. To confirm that the selected cell lines reflected the KRGS characteristics, we compared the expression levels of eight keratinization-related genes identified through GSEA in the CCLE dataset. Consistent with KRGS expression patterns, the expression of *ABCA12*, *PPL*, *CERS3*, and *IVL* was significantly lower in KRGS^low^ cell lines compared with KRGS^high^. Although the expression levels of *CNFN*, *SPRR2F*, *TMEM79*, and *SFN* did not show statistically significant differences, a similar trend of lower expression in KRGS^low^ was observed (Supplementary Fig. 5 A). To further validate the KRGS subgroups, we performed RT-PCR on the selected cell lines. The RT-PCR results showed a similar trend to those from the CCLE dataset, with significantly lower expression of *CNFN*, *SPRR2F*, *PPL*, *CERS3*, *TMEM79*, and *ABCA12* in KRGS^low^ cell lines compared with KRGS^high^ cell lines. Although the expression levels of *IVL* and *SFN* did not reach statistical significance, a similar pattern of lower expression was noted in KRGS^low^ cell lines (Supplementary Fig. 5B).

We then evaluated the correlation between KRGS subgroups and radiation response in the selected cell lines. Consistent with the clinical findings from the TCGA dataset, where KRGS^high^ was associated with radiation sensitivity, KRGS^high^ cell lines exhibited increased sensitivity to radiation compared with KRGS^low^ cell lines. Notably, SNU46 exhibited the highest resistance to radiation, followed by progressively lower resistance in HSC2, YD8, SCC9, SNU1066, and CAL27 cell lines (Fig. 3B). We next sought to investigate the mechanisms underlying the differential radiation resistance observed between KRGS^low^ and KRGS^high^ cell lines. Previous studies have shown that increased keratinization promotes cornification and contributes to reduced radiation resistance through cell death mechanisms [32]. To explore this, we performed a cornified envelope assay and counted the number of cornified envelopes in HNSCC cells. Although no significant difference was observed in the number of cornified envelopes in the absence of radiation (Supplementary Fig. 6 A), upon irradiation, the number of cornified envelopes increased in KRGS^high^ cell lines, whereas no such upregulation was observed in KRGS^low^ cell lines. Furthermore, there were differences observed between these two subgroups of cell lines (Supplementary Fig. 6B).

Given that KRGS^low^ cell lines exhibited strong radiation resistance and a lower number of cornified envelopes, we aimed to induce keratinization using ATRA and evaluate its effects on cornified envelope formation and RT efficacy. ATRA has been reported to induce keratinization by regulating keratinocyte proliferation and differentiation through retinoic acid receptor-mediated signaling [33–36]. To assess ATRA’s cytotoxicity, we first conducted an MTT assay and determined that ATRA exhibited cytotoxic effects at higher concentrations, with a 50% toxic concentration (TC50) of approximately 100 μM/L. On the basis of its impact on cell viability, we selected 10 μM/L as the treatment concentration (Supplementary Fig. 7). To evaluate the radiosensitizing effect of ATRA, KRGS^low^ cell lines were treated with 10 μM/L ATRA and irradiated at 2, 4, and 8 Gy. As shown by the CFA, ATRA-treated cells exhibited increased radiation sensitivity compared with control cells in HSC2, YD8, and SNU46 cell lines (Fig. 3C). We then quantified the number of cornified envelopes in KRGS^low^ cell lines following ATRA treatment with irradiation. ATRA-treated or irradiated cells exhibited a modest increase in cornified envelope formation compared with the control cells. When ATRA was combined with IR treatment, there was a significant increase in the number of cornified envelopes in all three KRGS^low^ cell lines compared with either ATRA or IR treatment, respectively. (Fig. 3D). We further assessed the expression of eight keratinization-related genes in these cell lines. In HSC2 cells, ATRA and IR co-treatment significantly increased the expression of *SPRR2F*, *IVL*, *ABCA12*, *CNFN*, and *CERS3* compared with either ATRA or IR treatment. However, no changes were observed in the mRNA levels of SFN and TMEM79. In YD8 cells, ATRA and IR co-treatment induced the upregulation of *SPRR2F*, *IVL*, *ABCA12*, *CNFN*, *SFN*, and *CERS3* compared with ATRA or IR treatment, but did not significantly affect *TMEM79* expression. In SNU46 cells, co-treatment with ATRA and IR significantly increased the expression of *SPRR2F*, *IVL*, *ABCA12*, and *SFN* compared with either ATRA or IR treatment, but did not affect the expression of *CNFN*, *CERS3*, or *TMEM79* (Supplementary Fig. 8). Collectively, our data suggests that KRGS^low^ cell lines are associated with radioresistance, which is linked to reduced cornified envelope formation and decreased expression of keratinization-related genes. Enhancing keratinization through ATRA treatment may serve as a potential strategy to overcome radioresistance in KRGS^low^ cell lines by promoting cornified envelope formation.

### Upregulation of keratinization increases radiosensitivity of radioresistant cells

**Fig. 4:**
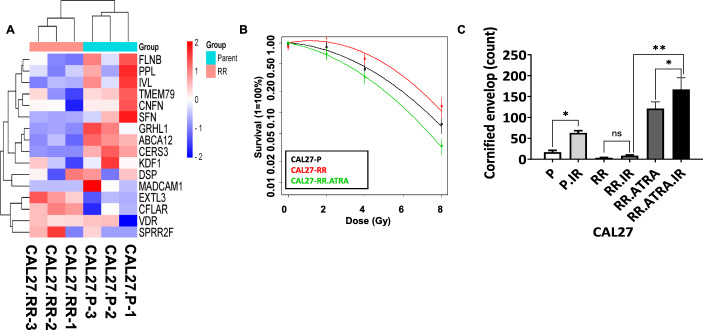
Efficacy evaluation of combined keratinization activation and IR in radioresistant HNSCC cell lines. **A** Heatmap illustrating the expression patterns of 16 KRGS genes in CAL27-P and CAL27-RR RNA sequencing data. Red indicates high expression levels, while blue represents low expression levels. **B** CFA demonstrating increased radiation sensitivity in ATRA-treated CAL27-RR cells to IR over a range of 0 to 8 Gy, as depicted in the log–linear plot. Each point represents the means of three replicates. Statistical significance was determined by ANOVA. Plating efficiency was higher than 12%. **C** Cornified envelope assay in CAL27-P and CAL27-RR cells. CAL27-RR cells were treated with either ATRA, IR, or ATRA with IR. Cornified envelopes were counted under a microscope. The *x*-axis represents the treatment groups: Con (untreated control), ATRA (ATRA treatment), IR (irradiation), and ATRA.IR (combined ATRA and irradiation treatment). The *y*-axis indicates the number of cornified envelopes. Error bars represent the SD. Statistically significant were determined unpaired Student’s *t*-test. *p < 0.05; **p < 0.01; ***p < 0.001

To further explore the impact of keratinization upregulation on radiation response efficacy in radioresistant cells, we utilized the CAL27-RR cell line, which was established in previous studies [37]. First, we compared the KRGS gene expression between the parental CAL27 (CAL27-P) cells and the radioresistant CAL27-RR cells using an RNA sequencing dataset. CAL27-P and CAL27-RR cells each had three independent RNA sequencing datasets for analysis. Heatmap analysis demonstrated a characteristic pattern in CAL27-RR cells, where 11 KRGS genes were significantly downregulated, while 5 KRGS genes were significantly upregulated (Fig. 4A). Subsequently, CFA was conducted to assess the association between RT efficacy and keratinization activation in CAL27-RR cells. ATRA significantly enhanced the sensitivity of CAL27-RR cells to irradiation (Fig. 4B). Additionally, the enhanced efficacy of RT was associated with an increase in the number of cornified envelope cells. Irradiation of CAL27-P cells resulted in a significant elevation in cornified envelope formation, with levels approximately three times higher than those observed in non-irradiated controls. In contrast, no significant difference was observed between non-irradiated and irradiated CAL27-RR cells. However, co-treatment with ATRA and irradiation induced a further increase in envelope formation compared with irradiated CAL27-RR cells. The extent of cornified envelope formation was significantly higher than that induced by ATRA as a single agent (Fig. 4C). These trends in cornified envelope formation were consistent with the mRNA expression levels of the eight keratinization-related genes in KRGS. The mRNA levels of these eight genes were significantly upregulated in irradiated CAL27-P cells compared with non-irradiated CAL27-P cells. In contrast, no significant changes in mRNA expression levels were observed between non-irradiated and irradiated CAL27-RR cells. However, treatment with ATRA in combination with irradiation led to a dramatic increase in the mRNA expression levels of these eight genes in CAL27-RR cells compared with ATRA or IR treatment. (Supplementary Fig. 9). These results suggest that keratinization activation by ATRA in combination with IR promotes cornified envelope formation through the upregulation of keratinization-related genes, thereby enhancing radiosensitivity in radioresistant HNSCC cells.

### Induction of cell death through combination of keratinization activation and irradiation via increased IVL expression in HNSCC cells

In squamous cell carcinoma, the cornified envelope, a form of programmed cell death, is formed through the desquamation process as basal cells migrate from the basal lamina to the granular layer, ultimately adopting a cornified state [35]. In this process, IVL plays a crucial role in the final stages of cornification (Supplementary Fig. 10) [36]. Given that IVL is a key regulator of cell death during keratinization, we performed Western blot analysis to determine whether ATRA-induced cell death was specifically mediated through IVL regulation, rather than through other apoptosis- or necrosis-related mechanisms. For this purpose, we examined the expression levels of apoptosis markers (caspase-3, MCL1), a necrosis marker (MLKL), and a proliferation marker (Ki-67) in HNSCC cells (SNU46, YD8, HSC3, and CAL27-P, RR cells) treated with ATRA and/or irradiation. The results indicated that treatment with ATRA and IR in the four HNSCC cell lines did not lead to a significant alteration in the expression levels of caspase-3, MCL1, MLKL, or Ki-67 when compared with nontreated control cells, IR-treated cells, and ATRA-treated cells, respectively. However, IVL expression was significantly upregulated in response to ATRA combined with IR in these four cell lines, in comparison with nontreated control cells, IR-treated cells, and ATRA-treated cells, respectively (Supplementary Fig. 11). These results notably suggest that, in combination with irradiation, ATRA induces cell death in a keratinization-dependent manner through the upregulation of IVL expression, independent of classical apoptosis or necrosis pathways.

### Integrin α1-mediated cell death induced by ATRA and IR in HNSCC cells

**Fig. 5:**
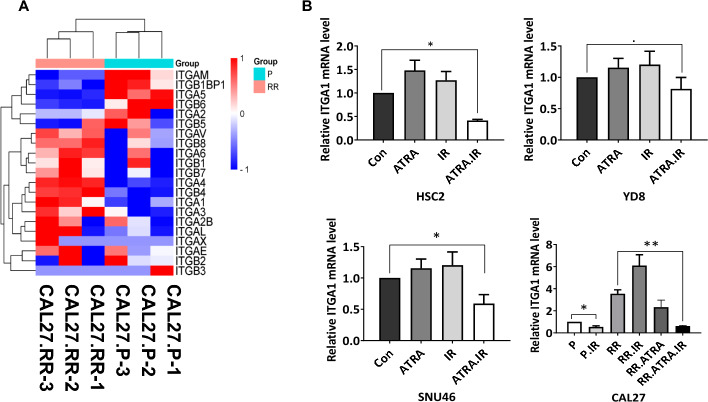
Upregulation of integrin α1 following combined ATRA and IR treatment in HNSCC cells. **A** Heatmap illustrating the expression patterns of 20 integrin GO genes in CAL27-P and CAL27-RR RNA sequencing data. Red indicates high expression levels, while blue represents low expression levels. **B** RT-PCR showing a decreased relative expression of integrin α1 in ATRA with IR-treated CAL27-RR cells compared with the untreated control. Cells were treated with ATRA for 24 h. The *x*-axis represents the treatment groups: Con (untreated control), ATRA (ATRA treatment), IR (irradiation), and ATRA.IR (combined ATRA and irradiation treatment). The *y*-axis shows relative ITGA1 mRNA expression levels normalized to the control. Data are presented as mean ± SD, and statistical significance was determined using Student’s *t*-test. *p < 0.05; **p < 0.01; ***p < 0.001

To explain the mechanisms by which ATRA combined with IR leads to a substantial increase in cornified envelope formation and cell death in HNSCC cells, we hypothesize that this increase is likely correlated with the modulation of integrin signaling. Previous studies have shown that integrin signaling can be activated by various factors, including chemotherapy and radiation [38]. Furthermore, integrin alpha 2 and beta 1 have been reported to be associated with the migration of keratinocytes [39]. ATRA has been reported to interact with integrin alpha 5 and beta 4 [40]. Consistent with these previous studies, our aforementioned GSEA of TCGA data also revealed an association with integrin-mediated signaling mechanisms in KRGS^low^ (Supplementary Fig. 4D). To further investigate the relationship between integrins and RT, we screened the 20 integrin genes presented by GO using RNA sequencing data from CAL27-P and CAL27-RR cells. Differentially expressed integrin genes between CAL27-P and CAL27-RR cells were identified (Fig. 5A). Seven differentially expressed genes (DEGs), including three downregulated and four upregulated, were confirmed (Supplementary Fig. 12 A, B).

Subsequently, we analyzed the mRNA expression levels of the seven integrin genes in HNSCC cells treated with ATRA, IR, or a combination of the two treatments. Upon individual treatment with either ATRA or IR, integrin α1 expression showed a modest increase compared with the control across KRGS^low^ cell lines. However, combined treatment with ATRA and IR resulted in a significant downregulation of integrin α1 expression in three KRGS^low^ cell lines. In CAL27-P cells, IR treatment slightly reduced integrin α1 expression relative to the control. In contrast, integrin α1 expression was elevated in irradiated CAL27-RR cells compared with untreated CAL27-RR cells. This increase in integrin α1 expression observed in CAL27-RR cells was dramatically reduced following combined treatment with ATRA and IR (Fig. 5B). Among the integrin subunits examined, integrin α1 consistently exhibited a uniform expression pattern across all cell lines in response to combined ATRA and IR treatment. The expression patterns of integrins α3, α4, α5, αM, β4, and β6 varied among the four cell lines. These findings suggest that integrin α1 may play a pivotal role in mediating the cell death mechanisms induced by the combination of ATRA and IR. Conversely the expression patterns of integrin α3, α4, α5, αM, β4, and β6 varied across the cell lines, indicating that the regulation of these integrins may be more complex and dependent on cell-specific factors (Supplementary Fig. 13).

### Enhancing keratinization sensitizes radiation therapy in xenograft models

**Fig. 6:**
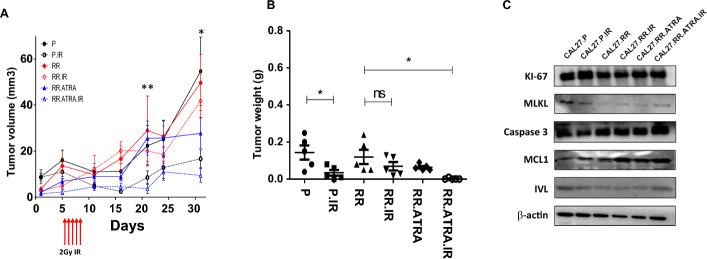
ATRA combined with IR enhances radiosensitivity in HNSCC xenograft mouse model. **A** Tumor volume was measured twice a week to assess the effects of the treatments over time. Statistical significance was determined using two-way ANOVA. The post hoc test was performed using Tukey’s method. *p < 0.05; **p < 0.01; ***p < 0.001. **B** Tumor weights were measured and compared across the indicated groups to evaluate the impact of ATRA and IR, both individually and in combination, on tumor growth. Statistical significance was determined using unpaired Student’s *t*-test. *p < 0.05; **p < 0.01; ***p < 0.001. **C** Protein expression levels in xenograft tissues from the CAL27.P (CAL27.P mouse group), CAL27.P IR (CAL27.P with IR treatment), CAL27.RR (CAL27.RR mouse group), CAL27.RR ATRA (CAL27.RR ATRA-treated mouse group), CAL27.RR IR (CAL27.RR IR-treated mouse group), and CAL27.RR ATRA.IR (CAL27.RR treated with both ATRA and IR) were analyzed by Western blot. β-Actin was included as an internal loading control to ensure equal protein loading across samples. The experiment was performed at least three times to confirm reproducibility

Given the results of in vitro keratinization activation with IR, which were implicated in the elevation of cell death in HNSCC, we further sought to determine whether keratinization activation and RT were sufficient to influence cell death in HNSCC in vivo. Therefore, we implanted 1 × 10^6^ CAL27-P cells and 1 × 10^6^ CAL27-RR cells into the right thighs of female nude mice [41]. The mice were randomly assigned to six groups (n = *5* per group): CAL27-P, CAL27-P with IR, CAL27-RR, CAL27-RR with IR, CAL27-RR with ATRA, and CAL27-RR with ATRA and IR. Three days after implantation of tumor, mice received a total of 10 Gy of irradiation, administered as 2 Gy per day for five consecutive days. Tumor growth was monitored for up to 33 days post-irradiation to assess the combined effect of ATRA and IR on tumor progression in vivo. CAL27-RR cells exhibited radioresistance compared with CAL27-P cells in the mouse model. Specifically, the CAL27-P group showed a reduction in tumor volume following IR treatment, whereas no significant difference in tumor volume was observed between the CAL27-RR group and the CAL27-RR with IR group. Remarkably, the mice treated with both ATRA and IR exhibited a significant reduction in tumor volume compared with the CAL27-RR group. However, no significant difference in tumor volume was observed between the CAL27-RR with ATRA group and the CAL27-RR group (Supplementary Fig. 14 A). Furthermore, tumor progression in the CAL27-RR group treated with ATRA and IR was significantly inhibited compared with the other five groups (Fig. 6A). After 33 days, the mice were euthanized, and tumor volume and weight were measured. Tumor weight was significantly decreased in the CAL27-P with IR group compared with CAL27-P. However, no significant differences were observed among the CAL27-RR, CAL27-RR with IR, and CAL27-RR with ATRA groups. The combination of ATRA and RT significantly decreased tumor weight compared with these three CAL27-RR groups. These results demonstrate that combined treatment with ATRA and RT effectively inhibited tumor growth in the mouse model (Fig. 6B). Furthermore, body weight changes were used as reliable indicators to assess the toxicity of the treatment. No significant changes in body weight were observed in the BALB/c mice throughout the 33-day experiment (Supplementary Fig. 14B).

Next, we evaluated whether the tumor growth inhibition mechanism is mediated by IVL regulation. For this purpose, we performed Western blot and IHC analyses using mouse tissue. As shown in the IHC results, the CAL27-P with IR group exhibited upregulation of IVL staining intensity compared with the CAL27-P group. The CAL27-RR and CAL27-RR with IR groups showed minimal IVL staining intensity. In the CAL27-RR with ATRA group, IVL staining intensity was slightly increased, while strong positive IVL staining was observed in the CAL27-RR with ATRA and IR group (Supplementary Fig. 15). Next, we examined the expression of cell death-related proteins across the six groups. The results showed a significant increase in IVL expression in the CAL27-RR with ATRA and IR group compared with ATRA or IR treatment. There were no statistically significant changes in the expression levels of caspase-3, MCL1, MLKL, and Ki-67 in the CAL27-RR cells treated with ATRA and IR, when compared with cells treated with either ATRA or IR (Fig. 6C, Supplementary Fig. 16) Together, our data suggest that keratinization induced by ATRA in combination with IR likely enhances the RT response in HNSCC in vivo.

### Depiction of KRGS subgroups in scRNA-seq analysis

**Fig. 7:**
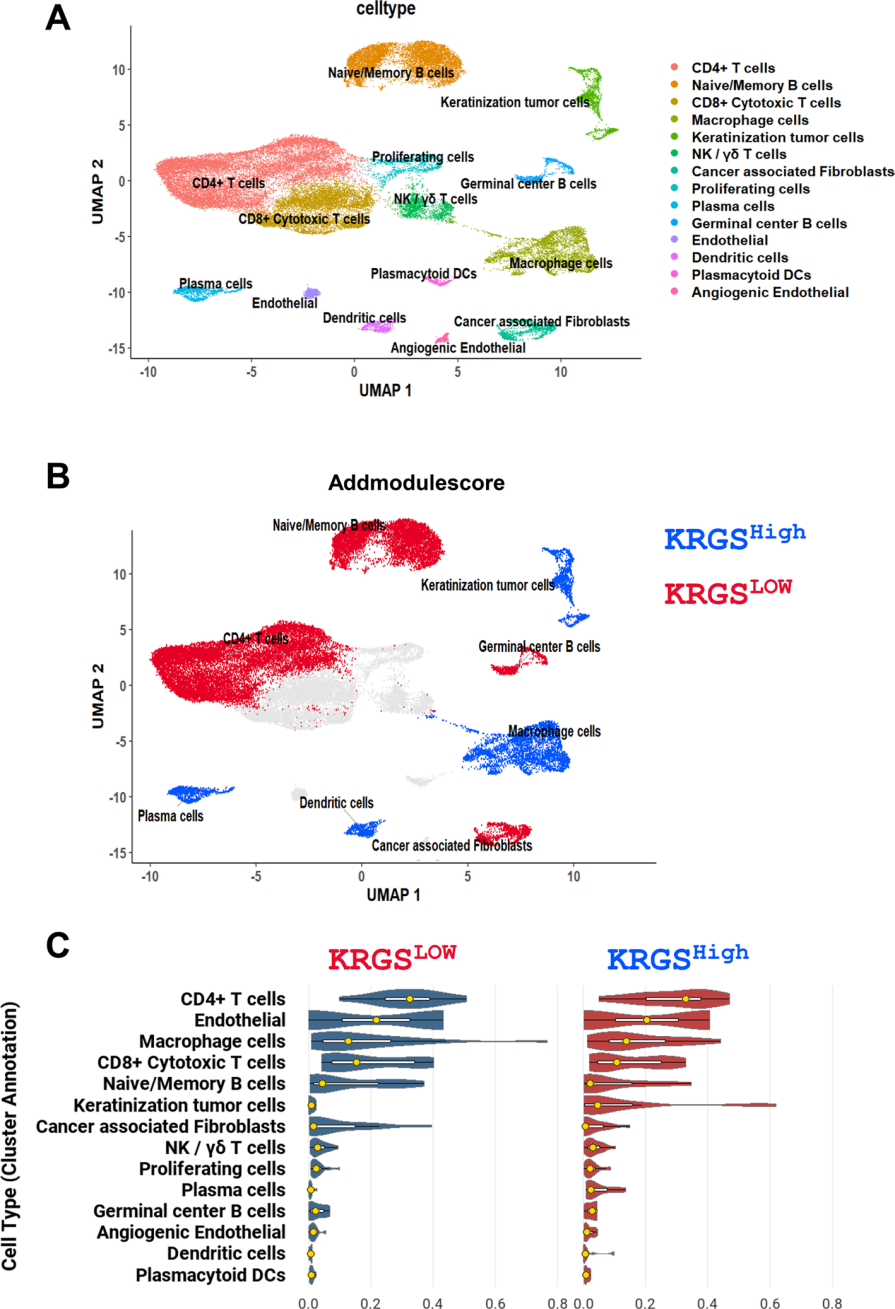
Single-cell characterization of KRGS subgroups. **A** UMAP plot displaying the distribution of cells from the GSE140042 dataset, revealing 14 distinct cell clusters following quality control and batch correction. **B** UMAP plot showing the distribution of KRGS subgroups across all cell types. Cells were divided into two subpopulations on the basis of the expression score of the KRGS signature. The AddModuleScore function was used to calculate KRGS activity in individual cells. Red represents KRGS^low^, and blue represents KRGS^high^. **C** Violin plot showing the KRGS module scores across all 14 identified clusters between KRGS^low^ and KRGS^high^

Keratinization represents the first line of defense against external insults, serving as both a physical and biochemical barrier. Owing to this role, it significantly influences the local immune environment by shaping immune cell infiltration and activation [42]. Previous studies have reported that increased expression of keratinization-related genes is associated with enhanced immune responses, suggesting a potential role of epithelial differentiation in modulating the tumor-immune microenvironment [43]. To investigate the association between immune responses and KRGS subgroups, we applied the CIBERSORT algorithm to estimate the relative abundances of 19 different immune cell types in KRGS^low^ and KRGS^high^ of KRGS from the TCGA HNSCC dataset. Macrophages M2 and resting memory CD4^+^ T cells account for a larger proportion in KRGS^low^ compared with KRGS^high^. In contrast, KRGS^high^ shows a higher proportion of neutrophils compared with KRGS^low^ (Supplementary Fig. 17). Then, to better understand the immune ecology between KRGS^low^ and KRGS^high^, we performed scRNA-seq analysis. The GSE140042 dataset was dimensionally reduced, clustered into 14 cell types, and annotated accordingly (Fig. 7A). Next, the AddModuleScore function was used to evaluate the activity levels of KRGS^low^ and KRGS^high^. KRGS^high^ was significantly associated with keratinization tumor cells macrophage, plasma, and dentritic cells as shown in the UMAP and violin plots. KRGS^low^ was associated with CD4^+^ T, naive/memory B, cancer-associated fibroblast, and germinal center B cells (Fig. 7B, C). Interestingly, the KRGS^high^ group predominantly consisted of adaptive immune components, including plasma cells, dendritic cells, and macrophages, which are associated with active antitumor immune responses. In contrast, the KRGS_low_ group was mainly enriched with stromal and naïve immune cell populations, such as cancer-associated fibroblasts, naïve/memory B cells, and CD4^+^ T cells, which contribute to extracellular matrix remodeling and an immunosuppressive tumor microenvironment. These results suggest that KRGS^low^ and KRGS^high^, which are associated with prognosis, exhibit markedly distinct characteristics across immunological contexts.

## Discussion

Recent studies have made efforts to identify distinct molecular subtypes within HNSCC that correlate with prognosis to offer promising avenues for personalized treatment approaches [44–47]. Despite these efforts, the clinical application of these molecular subtypes has not yet been fully realized, primarily owing to the need for further validation in diverse patient populations and the lack of sufficient in vitro and vivo validation before clinical implementation. In light of these challenges, we developed and validated a gene expression signature in various cohorts of patients with HNSCC, focusing this study on functional investigations through in vitro and in vivo models to facilitate clinical implementation. Our efforts led to the discovery of a novel KRGS, which was successfully validated in multiple large, publicly available datasets. In addition, our KRGS proved to be an extremely robust factor in predicting the prognosis of HNSCC, outperforming all previously reported histopathological features. We observed that the survival outcomes for KRGS^low^ were poorer than those for KRGS^high^ across four publicly accessible cohorts. Similarly, in our KHU cohort, KRGS validation confirmed that KRGS^low^ had worse clinical outcomes than KRGS^high^. Furthermore, KRGS^low^ was significantly associated with a poorer response to RT. Considering that RT serves as a primary treatment modality in HNSCC, playing a critical role in preserving both functional and structural integrity [48], we presented the results on the role of KRGS in predicting RT efficacy through in vitro and in vivo studies. In this study, we demonstrate that activation of keratinization through ATRA-induced upregulation of the cornified envelope enhances the response to RT. This upregulation of the cornified envelope was notably associated with keratinization-related genes within KRGS. Additionally, radioresistant HNSCC cells exhibited reduced radioresistance following treatment with ATRA in combination with IR, which induced the cornified envelope and upregulated eight keratinization-related genes. These enhancements in radiosensitization, associated with the downregulation of integrin α1 expression by ATRA in combination with IR, also led to an increase in IVL expression, a major regulator of terminal differentiation during cornification, ultimately resulting in cell death in HNSCC. Additionally, ATRA in combination with IR dramatically decreased mouse tumor volume. These decreases were also induced in an IVL expression-dependent manner, suggesting that keratinization activation with IR could be a promising strategy to overcome radioresistance in HNSCC. Furthermore, single-cell analysis revealed that KRGS^low^ was associated with CD4^+^ T, naive/memory B, cancer associated fibroblast, and germinal center B cells, whereas KRGS^high^ was related to keratinization tumor cells macrophage, plasma, and dendritic cells. These findings suggest that the prognostic differences between KRGS^low^ and KRGS^high^ may be attributed to distinct immune responses.

Keratinization refers to the process by which squamous cells, such as those in squamous cell carcinoma, undergo differentiation to form keratin, leading to cell death and the accumulation of keratin proteins [49, 50]. This process begins in the basal layer of the epithelium and progresses as the cells move upward toward the stratum corneum, where they eventually become fully keratinized [13]. In cancer, specific keratin patterns are largely maintained in tumor cells and are associated with their respective cells of origin, serving as diagnostic tumor markers [18, 51]. Previous studies have reported that keratinization, as assessed by histological features, is correlated with poor prognosis in various cancers, including nasopharyngeal carcinoma (NPC), lung, breast, and oral squamous cell carcinoma (OSCC) [19, 20, 52–54]. In HNSCC, keratinized squamous cell carcinoma has been linked to poor prognosis, with hyperkeratinization acting as a significant factor in the tumorigenesis of HNSCC [19, 55]. Conversely, keratinization suppressed migration and invasion in HNSCC, with increase in the cornified envelope [56, 57]. Furthermore, keratinization expression were reported to change during metastatic progression of breast cancer [58]. Keratinization is influenced by the environment and cellular diversity, which is considered a key factor in increasing tumor heterogeneity [59]. As a result, it is crucial to identify a molecular gene signature related to keratinization that quantifies disease characteristics and reflects heterogeneity for personalized patient treatment. In this study, 16 KRGS were identified and found to predict survival and RT response outcomes in HPV-negative HNSCC. Patients in KRGS^high^ with high expression of the 16 genes showed better prognosis than those in KRGS^low^, which was accompanied by enhanced cell death with IR. The mechanism of KRGS-related cell death follows the progression of differentiation from the basal layer, where keratinocyte differentiation factor 1 (KDF1) and caspase-8 and Fas-associated death domain-like apoptosis regulator (CFLAR) regulate proliferation and apoptosis [60, 61], through the stratum spinosum, where DSP, FLNB, PPL, VDR, GRHL1, MADCAM1, and EXTL3 coordinate cytoskeletal organization, adhesion, and immune regulation [32, 62, 63]. In the stratum granulosum, TMEM79 and SFN modulate filaggrin processing, lipid barrier formation, and cell cycle arrest [64]. Finally, in the cornified layer, SPRR2F, CNFN, CERS3, and ABCA12 establish the cornified envelope and lipid barrier, ensuring epidermal integrity [32, 63]. At the later stage of differentiation, IVL induces cornification-associated cell death, contributing to tumor suppression [65] (Supplementary Fig. 10). In this study, the combination of ATRA and IR induced cornified envelope formation through the downregulation of ITGA1 mRNA levels and enhanced the response to RT in HNSCC. This is consistent with previous studies reporting that high ITGA1 expression is associated with poor prognosis in HNSCC, and its inhibition suppresses metastasis and proliferation [66]. ITGA1 has been reported to form a heterodimer with ITGB1 and play a role in cell adhesion, signaling, and differentiation regulation, influencing keratinocyte proliferation and survival [67, 68]. Furthermore, we identified integrin genes (α3, α4, α5, αM, β4, and β6) associated with radioresistance across different HNSCC cell lines. The expression of these six integrin genes varied in response to ATRA, IR, and the combination of ATRA and IR, respectively. These findings indicate that the regulation of these integrins is highly complex and may depend on cell-specific factors. Given the role of integrins in cell adhesion and signaling, further studies are required to elucidate their precise relationship with keratinization and how this interaction influences cell fate and tumor progression in HNSCC.

Keratinization is a critical component of the tumor immune barrier. Generally, immune effector cells face challenges infiltrating solid tumors and often exhibit impaired functionality [42, 43]. Therefore, understanding the pivotal role of the immune system in cancer prognosis may provide insights to improve RT efficacy. Previous studies have shown that keratinization is strongly associated with immune responses [69]. Accordingly, we evaluated immune cell types in the two KRGS subgroups.

In the CIBERSORT analysis, KRGS^low^ showed higher proportions of M2 macrophages and resting memory CD4^+^ T cells compared with KRGS^high^. M2 macrophages contribute to tumor progression through immunosuppression, extracellular matrix (ECM) remodeling, and angiogenesis [70]. Resting memory CD4^+^ T cells, characterized by a hyporesponsive state, may reflect reduced antitumor immunity, potentially indicating T cell exhaustion or immune evasion within the tumor microenvironment [71]. In contrast, KRGS.^high^ showed higher proportions of neutrophils. Neutrophils and natural killer (NK) cells play crucial roles in early tumor elimination; neutrophils release cytotoxic molecules, while NK cells recognize and destroy tumor cells through stress-induced ligands [72].

Furthermore, single-cell analysis revealed distinct immune landscapes between the two groups. The KRGS^low^ subgroup predominantly contained CD4^+^ T cells, naïve/memory B cells, germinal center B cells, and cancer-associated fibroblasts, indicating an immunosuppressive tumor microenvironment. CD4^+^ T cells in HNSCC often include regulatory T cells that suppress cytotoxic activity, while excessive naïve or memory B cells may represent a dysfunctional humoral response [73, 74]. Disorganized germinal center B cells can secrete immunoregulatory cytokines that favor tumor progression [75], and CAFs further contribute to an immunosuppressive and fibrotic stroma through transforming growth factor (TGF)-β and interleukin (IL)−6 secretion and extracellular matrix remodeling [76, 77]. In contrast, the KRGS^high^ subgroup was characterized by a higher presence of keratinization tumor cells, macrophages, plasma cells, and dendritic cells, suggesting an immune-enriched tumor microenvironment. Notably, the increased keratinization feature in the KRGS^high^ group was consistent with the results from the KRGS. Macrophages in this context may promote antigen presentation and antitumor inflammation, while plasma cells contribute to antibody-mediated immunity within the tumor [78]. The increased presence of dendritic cells further suggests enhanced antigen presentation and T-cell priming, facilitating stronger immune surveillance [79, 80]. Together, these findings indicate that KRGS^high^ tumors maintain an active and coordinated antitumor immune response, which may underlie their greater radiosensitivity and favorable prognosis. Overall, the distinct immune cell compositions between KRGS^low^ and KRGS^high^ appear to drive their differential responses to radiotherapy and clinical outcomes in HNSCC.

A limitation of this study is that spatial characteristics of the tumor microenvironment were not captured in the current single-cell RNA-seq analysis. Future studies will incorporate spatial transcriptomic approaches, such as Visium HD, to better delineate the spatial context and cellular interactions within HNSCC.

In conclusion, we firmly propose that this novel 16 KRGS serves as a powerful and reliable prognostic biomarker for HPV-negative HNSCC. This signature consistently and reproducibly stratifies patients into two distinct molecular subgroups across multiple independent cohorts, underscoring its strong clinical applicability. Our findings clearly demonstrate that KRGS is a significant predictor of radiotherapy response and patient survival, highlighting its potential as a clinically actionable classifier. Moreover, we reveal that keratinization activation, through the combination of ATRA and IR, suppresses ITGA1 expression and overcomes radioresistance in HNSCC cells. Collectively, this study provides compelling evidence that KRGS bridges the molecular mechanisms of keratinization and radiation response, offering both a robust prognostic tool and novel therapeutic insights for future clinical translation.

## Supplementary Information

Supplementary Material 1: Figure 1. Consensus clustering and cluster stability assessment.CDF plots depicting the cumulative distribution functions of consensus matrices for *k* = 2, 3, 4, and 5, illustrating the stability of clustering solutions.Cluster tracking plot visualizing the assignment of tumor samplesacross different *k* values, with colors indicating distinct cluster memberships. This plot highlights the consistency of sample clustering across resolutions.Consensus clustering heatmap for the TCGA-HNSC cohort at *k* = 2, illustrating the consensus scores and cluster separation among samples.

Supplementary Material 2: Figure 2. Calibration of predicted and observed 3- and 5-year overall survival probabilities.Calibration plots comparing predicted and actual overall survival probabilities at 3- and 5-year follow-up. The bootstrapped calibration plot for 3- and 5-year overall survival prediction is shown. The black line represents the ideal fit, the blue line indicates nomogram-predicted probabilities, stars denote bootstrap-corrected estimates, and error bars represent the 95% confidence intervals of these estimates

Supplementary Material 3: Figure 3. Prognostic significance of the KRGS in relation to tumor stage.Kaplan–Meier plots for RFS of patients in KRGS_low_ and KRGS_high_ in advanced tumor stage.Kaplan–Meier plots for RFS of patients in KRGS_low_ and KRGS_high_ in early tumor stage. Statistical significance was assessed using the log-rank test.

Supplementary Material 4: Figure 4. Significantly enriched GO pathway analysis in KRGSlow.Significantly negatively enriched GO pathways of KRGS genes in KRGS_low_.Bubble plot of the top 20 negatively enriched pathways in KRGS_low_. The *x*-axis represents the enrichment score, and the bubble size indicates the number of genes. Color gradient from blue to red represents the *p*-value, with blue indicating low and red indicating high.Representative protein–protein interactionnetwork of eight keratinization-related genes from GSEA, visualized using Cytoscape. Nodes represent proteins, and edges indicate predicted interactions. Red-colored nodes highlight key proteins involved in keratinization.Significantly positively enriched GO pathways of KRGS genes in KRGS_low_.Bubble plot of the top 20 positively enriched pathways in KRGS_low_. The *x*-axis represents the enrichment score, and the bubble size indicates the number of genes. Color gradient from blue to red represents the *p*-value, with blue indicating low and red indicating high.

Supplementary Material 5: Figure 5. Expression profiles of keratinization-related genes in HNSCC cell lines.Expression levels of eight keratinization-related genes from CCLE data in six HNSCC cell lines. Statistical significance was determined unpaired Student’s *t*-test. *p < 0.05; **p < 0.01; ***p < 0.001 Expression levels of eight keratinization-related genes in six HNSCC cell lines were analyzed by RT-PCR

Supplementary Material 6: Figure 6. Assessment of cornified envelope formation in HNSCC cell lines.Cornified envelope assay showing cornified envelope formation in six cell lines without radiation exposure. Cornified envelopes were counted under a microscope. The *x*-axis represents HNSCC cell lines, and the *y*-axis represents the number of cornified envelopes. Error bars represent SD.Cornified envelope assay showing cornified envelope formation in six cell lines after radiation exposure. Cornified envelopes were counted under a microscope. The *x*-axis represents HNSCC cell lines, and the *y*-axis represents the number of cornified envelopes. Error bars represent SD.

Supplementary Material 7: Figure 7. Viability of ATRA in HNSCC. MTT assay results demonstrating the effect of ATRA on cell viability in HNSCC cells. Cells were treated with various concentrations of ATRA for 24 h. Values represent the mean ± SD of three independent experiments. Error bars represent SD. Cell viability was measured by reading absorbance at 570 nm using a microplate reader.

Supplementary Material 8: Figure 8. Relative mRNA expression levels of keratinization-related genes in KRGS_low_ cell lines. The *x*-axis indicates the analyzed genes, and the *y*-axis shows the relative mRNA levels normalized to control. Bar colors represent treatment groups as follows: gold for the untreated control, light gray for ATRA treatment, dark gray for irradiation, and black for the combined ATRA and irradiation treatment. Cells were treated with ATRA for 24 h, and statistically significant were determined unpaired Student’s* t*-test. *p < 0.05; **p < 0.01; *p < 0.001. Data are presented as mean ± SD.

Supplementary Material 9: Figure 9. Relative mRNA expression levels of keratinization-related genes in CAL27-P and CAL27-RR cells. The *x*-axis represents the experimental groups: P, P.IR, RR, RR.IR, RR.ATRA, and RR.ATRA.IR. The *y*-axis shows the relative mRNA expression levels normalized to the untreated control. Cells were treated with ATRA for 24 h. Data are presented as mean ± SD, and statistically significant were determined unpaired Student’s *t*-test. *p < 0.05; **p < 0.01; *p < 0.001.

Supplementary Material 10: Figure 10. Schematic visualization of keratinization progressing to cornification in relation to KRGS genes

Supplementary Material 11: Figure 11. Cell death mechanism induced by the combination of keratinization activation and IR through IVL expression in HNSCC. Protein levels in HNSCC cell lines were determined by Western blot. β-Actin was used as an internal loading control. The experiment was performed at least three times

Supplementary Material 12: Figure 12. Differential expression of integrin genes between CAL27-P and CAL27-RR cells.Volcano plot of differentially expressed integrin genes between CAL27-P and CAL27-RR in RNA sequencing data. The *y*-axis represents the *p*-value, and the *x*-axis represents the fold change.The six significantly differentially expressed integrin genes between CAL27-P and CAL27-RR

Supplementary Material 13: Figure 13. Expression changes of integrin genes upon ATRA treatment and IR exposure. mRNA expression levels of six integrin genesin KRGS_low_, CAL27-P, and CAL27-RR HNSCC cell lines were analyzed by RT-PCR. Experiments were performed at least three times. Cells were treated with ATRA for 24 h. The *x*-axis represents the treatment groups: Con, ATRA, IR, and ATRA.IR. The *y*-axis shows relative mRNA expression levels normalized to the control. Data are presented as mean ± SD.

Supplementary Material 14: Figure 14. Impact of treatment on tumor growth and systemic effects in mouse models.Tumor-bearing mice were categorized into two groups based on tumor type: CAL27-P and CAL27-RR. Mice with CAL27-P tumors received either irradiationor no treatment, mice with CAL27-RR tumors were treated with ATRA, IR, a combination of two agents, or left untreated as controls. Representative photographs of dissected tumors from each experimental group are presented. Mouse body weights were recorded at consistent intervals throughout the experiment to monitor any treatment-related systemic toxicity or adverse effects. Mouse body weights were measured twice a week

Supplementary Material 15: Figure 15. IHC analysis of IVL expression in xenograft tumor tissues.Representative IHC staining images of xenograft tumor tissues from the following mouse groups: CAL27.P, CAL27.P IR, CAL27.RR, CAL27.RR ATRA, CAL27.RR IR, and CAL27.RR ATRA.IREach column shows paired low-magnificationand high-magnificationviews of the same region.These images were analyzed to assess the intensity of IVL expression levels in response to the different treatment conditions. Image Jwas performed to measure intensity of IVL. Statistical significance of differences in IVL expression was determined using unpaired Student’s *t*-test. *p < 0.05; **p < 0.01; *p < 0.001.

Supplementary Material 16: Figure 16. Quantification of protein from western blot. Protein expression levels in xenograft tissues from the following mouse groups were quantified: CAL27.P, CAL27.P IR, CAL27.RR, CAL27.RR ATRA, CAL27.RR IR, and CAL27.RR ATRA.IR. The *x*-axis represents the different treatment groups, and the *y*-axis represents the intensity of protein quantification. Statistical significance was determined using an unpaired Student’s *t*-test. *p < 0.05; **p < 0.01; ***p < 0.001.

Supplementary Material 17: Figure 17. Estimation of immune cell proportions in KRGS subgroups. Proportions of 19 immune cell types, estimated using CIBERSORT were analyzed in HNSCC from the TCGA database, comparing KRGS_low_ and KRGS_high_ of the KRGS. Statistical significance was determined using an unpaired Student’s *t*-test. *p < 0.05; **p < 0.01; ***p < 0.001.

Supplementary Material 18.

Supplementary Material 19.

## Data Availability

The data that support the findings of this study are openly available in the following databases: TCGA database (https://xenabrowser.net/), Leipzig database, FHCRC database, and MDACC database (https://www.ncbi.nlm.nih.gov/gds), and CCLE database (https://portals.broadinstitute.org/ccle). The KHU database are available in Ref. [47].

## Abbreviations

HNC: Head and neck cancer
HNSCC: Head and neck squamous cell carcinoma
HPV: Human papillomavirus
AJCC: American Joint Committee on Cancer
OS: Overall survival
RT: Radiation therapy
TCGA: The Cancer Genome Atlas
GEO: Gene Expression Omnibus
IRB: Institutional review board
CCLE: Cancer Cell Line Encyclopedia
KRGS: Keratinization-related gene signature
CoxPH: Cox proportional hazards
GO: Gene Ontology
CDF: Cumulative distribution function
HR: Hazard ratio
CI: Confidence interval
BCCP: Bayesian compound covariate predictor
GSEA: Gene set enrichment analysis
FDR: False discovery rate
KCLB: Korea Cell Line Bank
ATCC: American Type Culture Collection
STR: Short tandem repeat
RPMI: Roswell Park Memorial Institute
DMEM: Dulbecco’s modified Eagle medium
CAL27-P: CAL27-parent
CAL27-RR: CAL27-radioresistant
FBS: Fetal bovine serum
PS: Penicillin–streptomycin
IR: Ionizing radiation
CFA: Colony-forming assays
cDNA: Complementary DNA
RIN: RNA integrity number
ATRA: All-*trans* retinoic acid
MTT: 3-(4,5-Dimethylthiazol-2-yl)-2,5-diphenyltetrazolium bromide
ANOVA: Analysis of variance
PVDF: Polyvinylidene fluoride
TBS: Tris-buffered saline
MLKL: Mixed lineage kinase domain-like protein
Caspase 3: Cysteine-aspartic protease
MCL1: Myeloid cell leukemia 1
Ki-67: Antigen Kiel 67
ITGA5: Integrin alpha-5
KHU: Kyung Hee university
FHCRC: Fred Hutchinson Cancer Research Center
MDACC: MD Anderson Cancer Center
RIPA: Radioimmunoprecipitation assay
EDTA: Ethylenediaminetetraacetic acid
BCA: Bicinchoninic acid
SDS: Sodium dodecyl sulfate
TBS-T: Tris-buffered saline with TWEEN
ECL: Enhanced chemiluminescence
ROC: Receiver operating characteristic
UMAP: Uniform Manifold Approximation and Projection
TC50: Toxic concentration
OSCC: Oral squamous cell carcinoma
NPC: Nasopharyngeal carcinoma
KDF1: keratinocyte differentiation factor 1
CFLAR: Fas-associated death domain-like apoptosis regulator
ECM: Extracellular matrix
NK: Natural killer
TGF: Transforming growth factor
IL: Interleukin
IVL: Involucrin
qRT-PCR: Quantitative real-time polymerase chain reaction
GAPDH: Glyceraldehyde-3-phosphate dehydrogenase
PBS: Phosphate-buffered saline
IHC: Immunohistochemistry
BSA: Bovine serum albumin
DAB: 3,3′-Diaminobenzidine
RFS: Recurrence-free survival
DSS: Disease-specific survival
SD: Standard deviation
DEG: Differentially expressed genes
