# Supplementary figures and images for: Keratinization-related gene signature predicting survival and response to radiation in patients with HPV-negative head and neck squamous cell carcinoma via regulation of cornification and integrin signaling
Source: Cell Mol Biol Lett. 2026 Jan 29;31:26. doi: 10.1186/s11658-025-00855-y (PMC12924225; doi:10.1186/s11658-025-00855-y)

Supplementary Figure 1

A

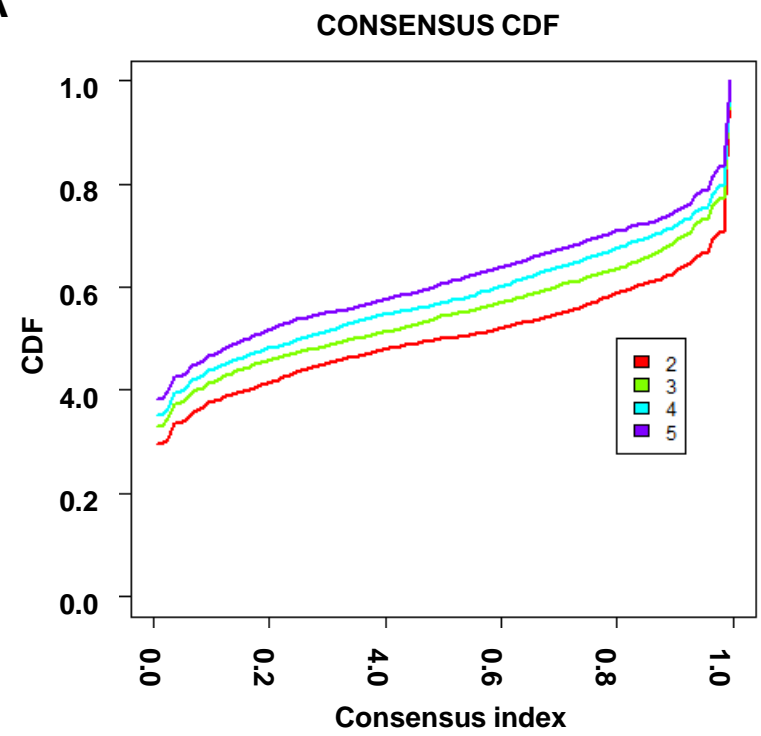

B

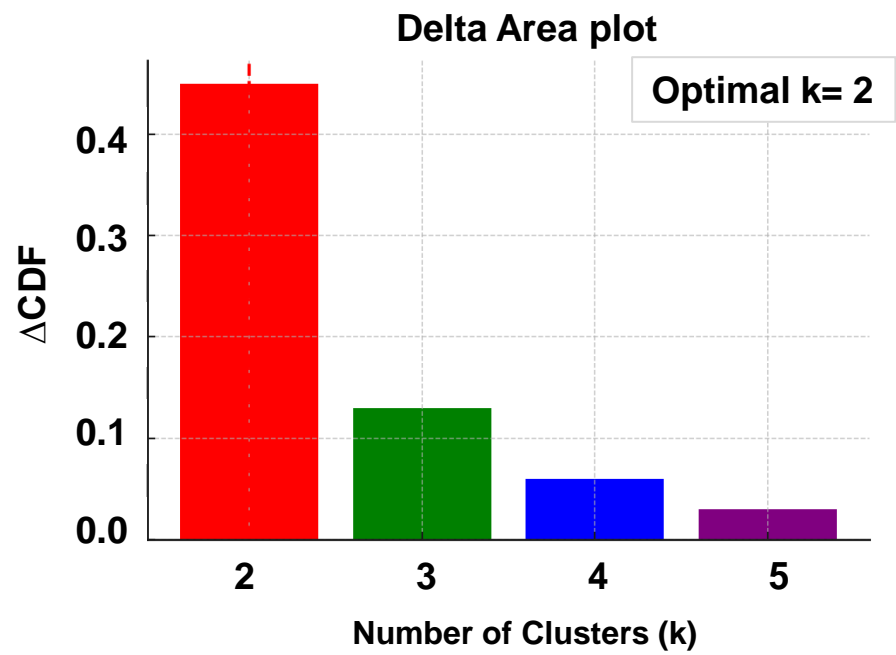

C

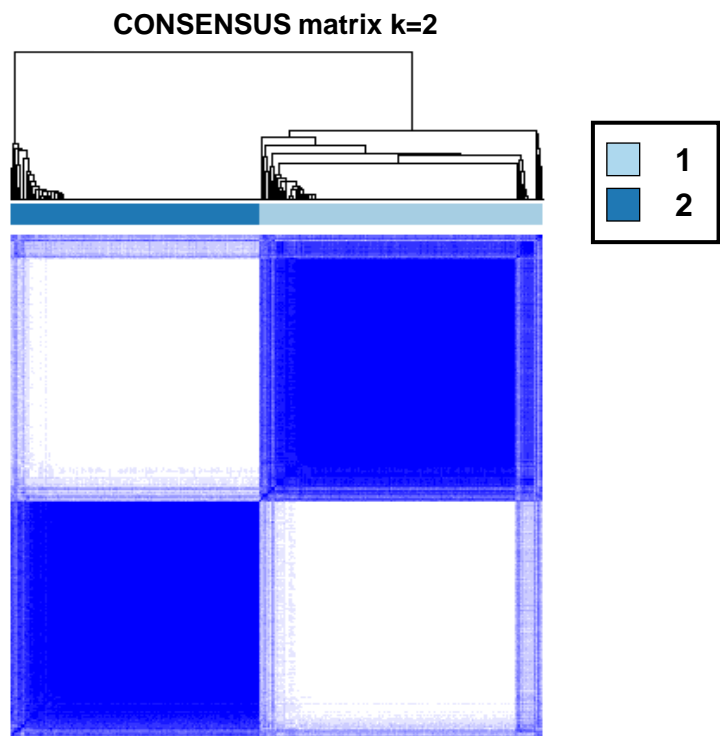

Supplement: Supplementary file 1 — Supplementary Material 1: Figure 1. Consensus clustering and cluster stability assessment.CDF plots depicting the cumulative distribution functions of consensus matrices for k = 2, 3, 4, and 5, illustrating the stability of clustering solutions.Cluster tracking plot visualizing the assignment of tumor samplesacross different k values, with colors indicating distinct cluster memberships. This plot highlights the consistency of sample clustering across resolutions.Consensus clustering heatmap for the TCGA-HNSC cohort at k = 2, illustrating the consensus scores and cluster separation among samples. [file 11658_2025_855_MOESM1_ESM.pdf]

Supplementary Figure 2

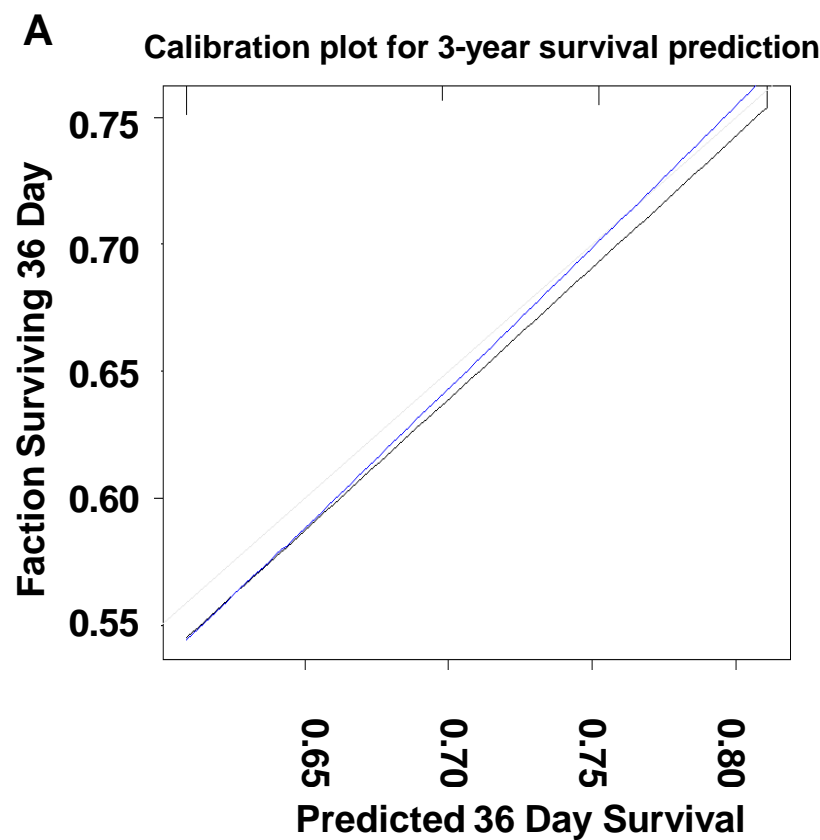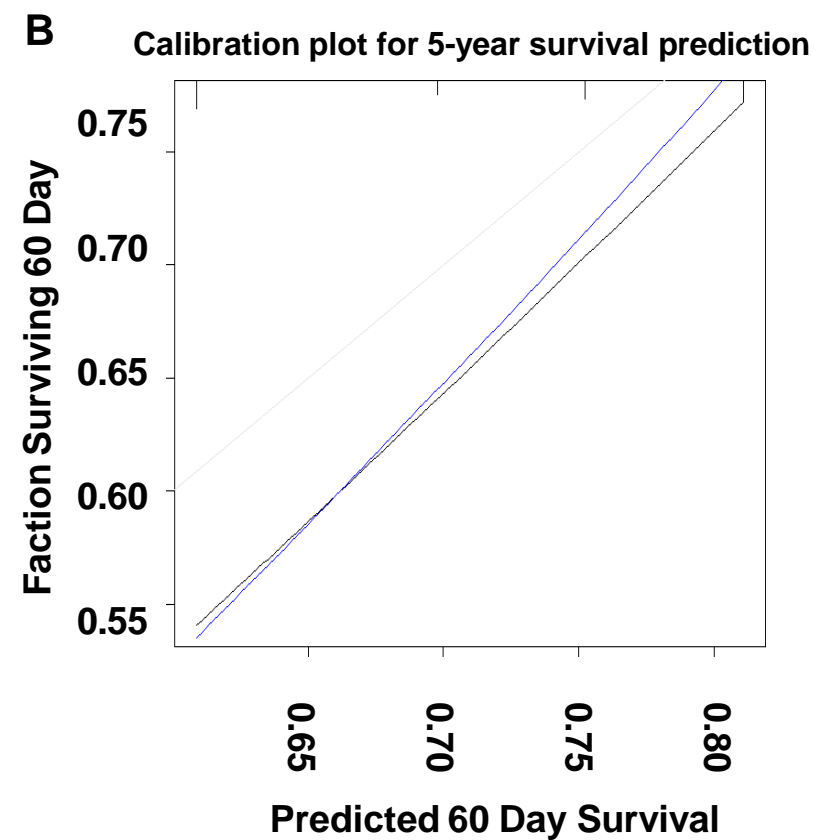

Supplement: Supplementary file 2 — Supplementary Material 2: Figure 2. Calibration of predicted and observed 3- and 5-year overall survival probabilities.Calibration plots comparing predicted and actual overall survival probabilities at 3- and 5-year follow-up. The bootstrapped calibration plot for 3- and 5-year overall survival prediction is shown. The black line represents the ideal fit, the blue line indicates nomogram-predicted probabilities, stars denote bootstrap-corrected estimates, and error bars represent the 95% confidence intervals of these estimates [file 11658_2025_855_MOESM2_ESM.pdf]

Supplementary Figure 3

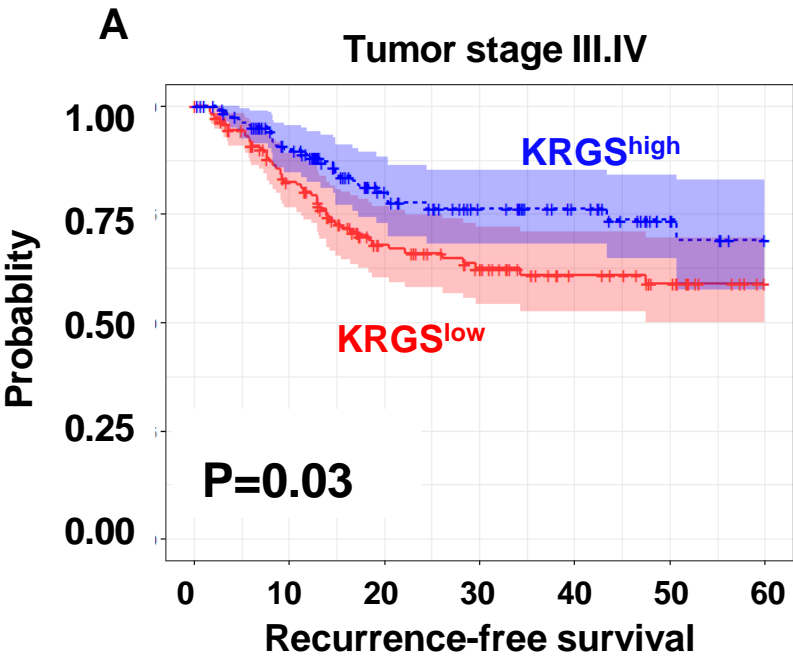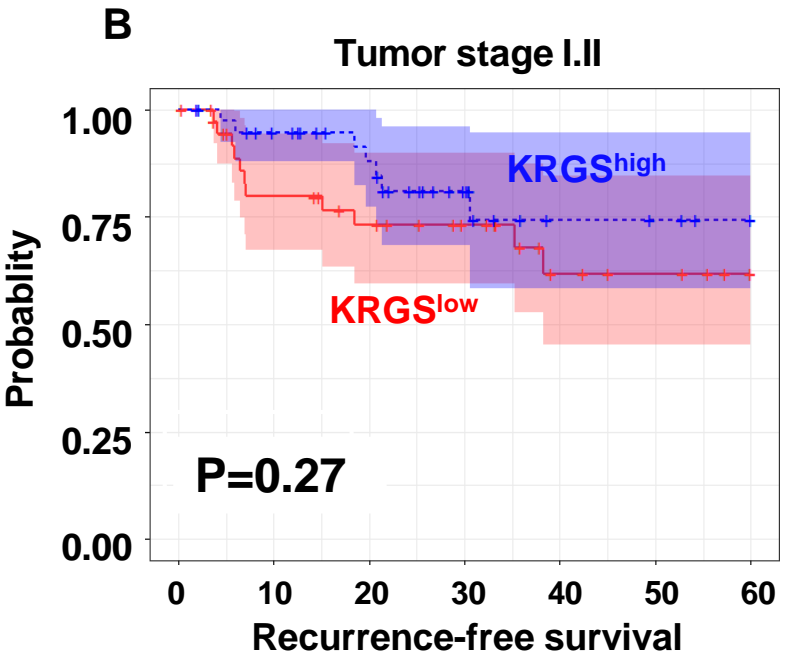

Supplement: Supplementary file 3 — Supplementary Material 3: Figure 3. Prognostic significance of the KRGS in relation to tumor stage.Kaplan–Meier plots for RFS of patients in KRGSlow and KRGShigh in advanced tumor stage.Kaplan–Meier plots for RFS of patients in KRGSlow and KRGShigh in early tumor stage. Statistical significance was assessed using the log-rank test. [file 11658_2025_855_MOESM3_ESM.pdf]

Supplementary Figure 4

A

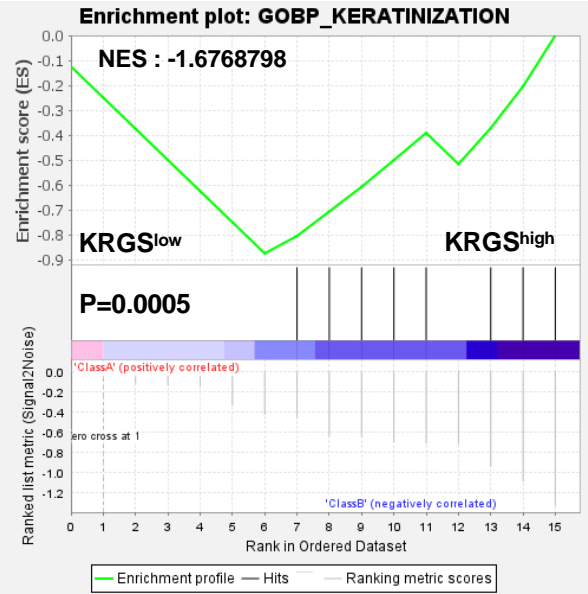

B

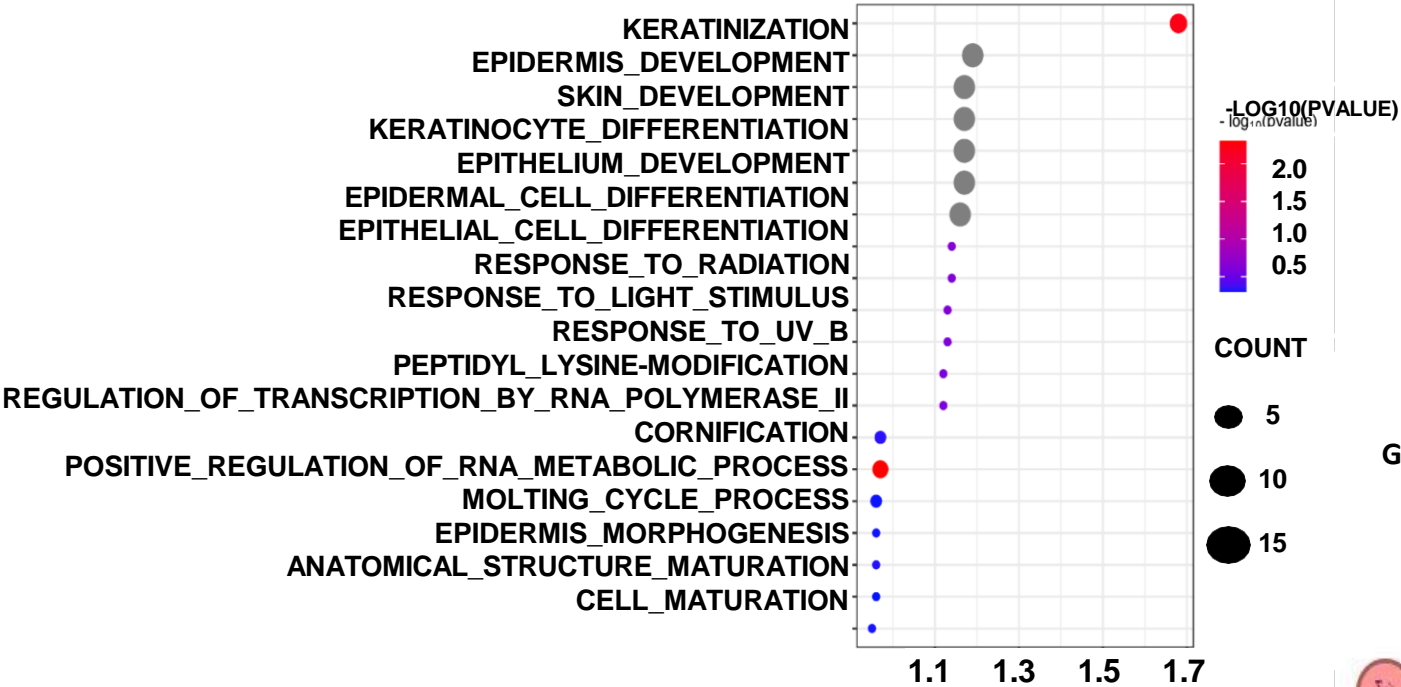

C

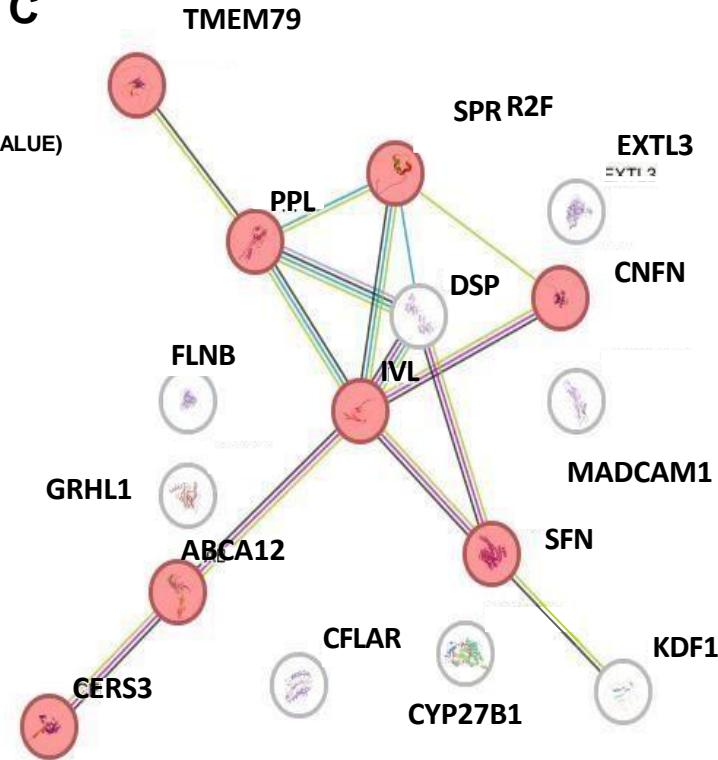

D

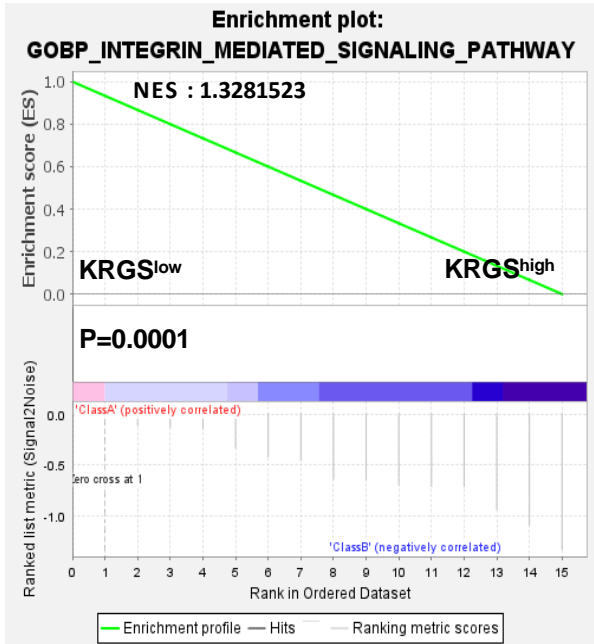

E

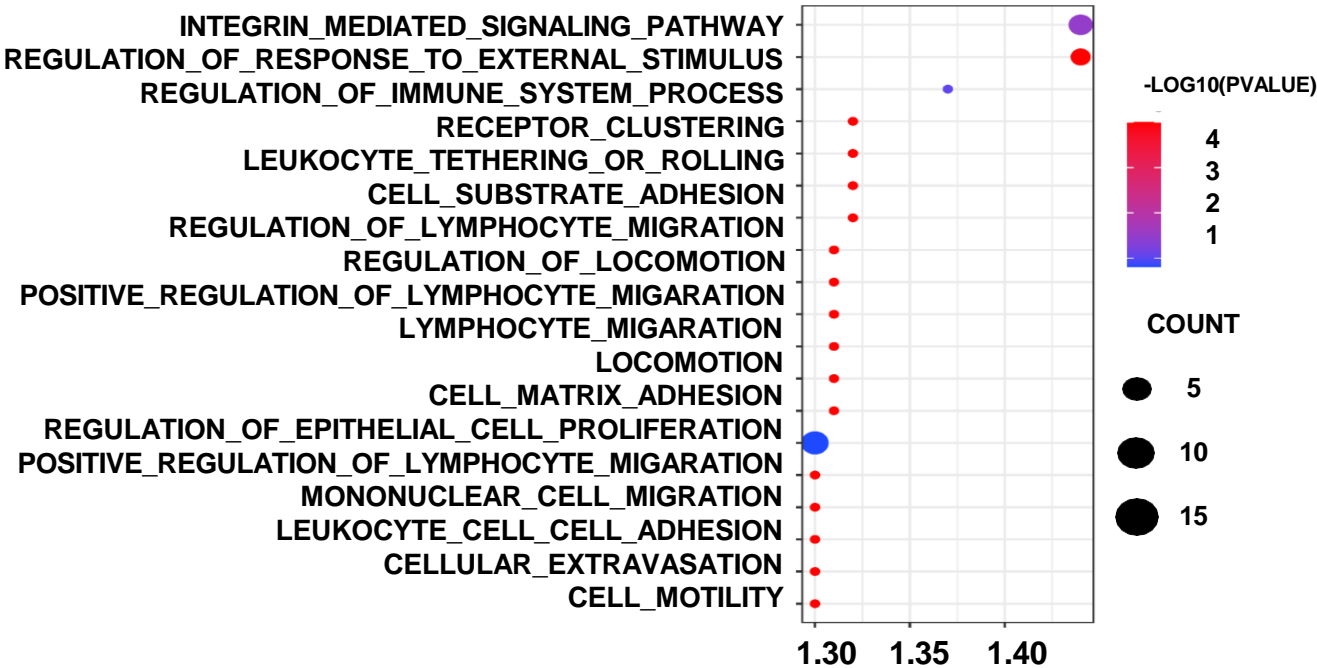

Supplement: Supplementary file 4 — Supplementary Material 4: Figure 4. Significantly enriched GO pathway analysis in KRGSlow.Significantly negatively enriched GO pathways of KRGS genes in KRGSlow.Bubble plot of the top 20 negatively enriched pathways in KRGSlow. The x-axis represents the enrichment score, and the bubble size indicates the number of genes. Color gradient from blue to red represents the p-value, with blue indicating low and red indicating high.Representative protein–protein interactionnetwork of eight keratinization-related genes from GSEA, visualized using Cytoscape. Nodes represent proteins, and edges indicate predicted interactions. Red-colored nodes highlight key proteins involved in keratinization.Significantly positively enriched GO pathways of KRGS genes in KRGSlow.Bubble plot of the top 20 positively enriched pathways in KRGSlow. The x-axis represents the enrichment score, and the bubble size indicates the number of genes. Color gradient from blue to red represents the p-value, with blue indicating low and red indicating high. [file 11658_2025_855_MOESM4_ESM.pdf]

Supplementary Figure 5

A

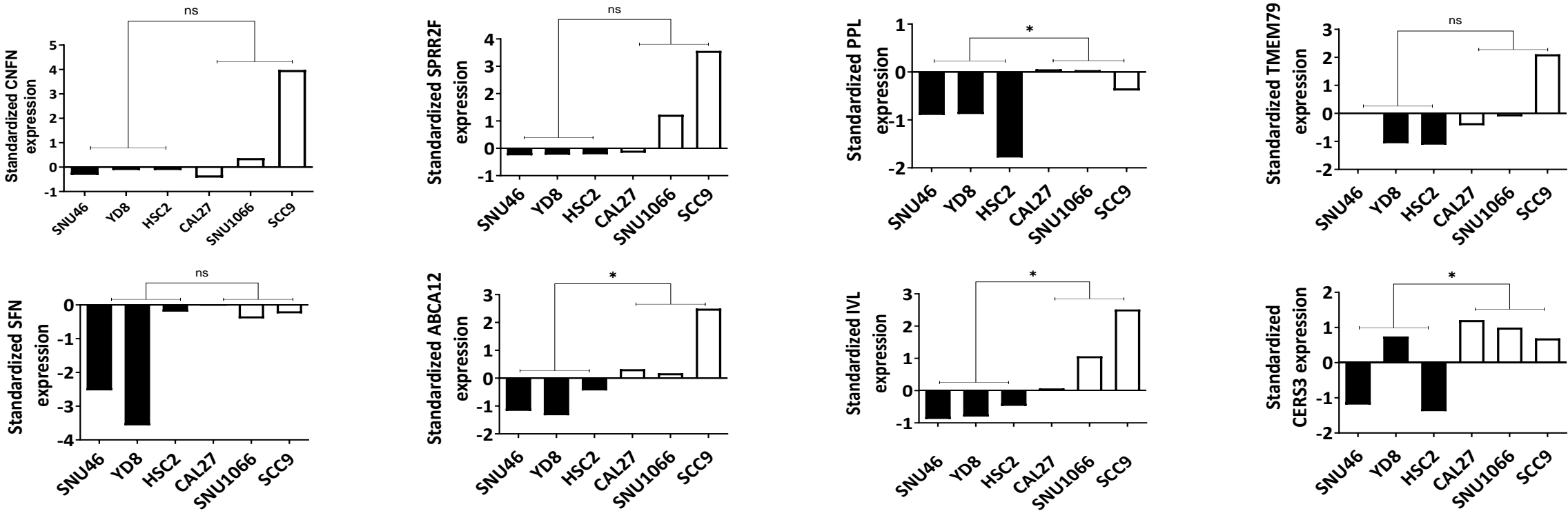

B

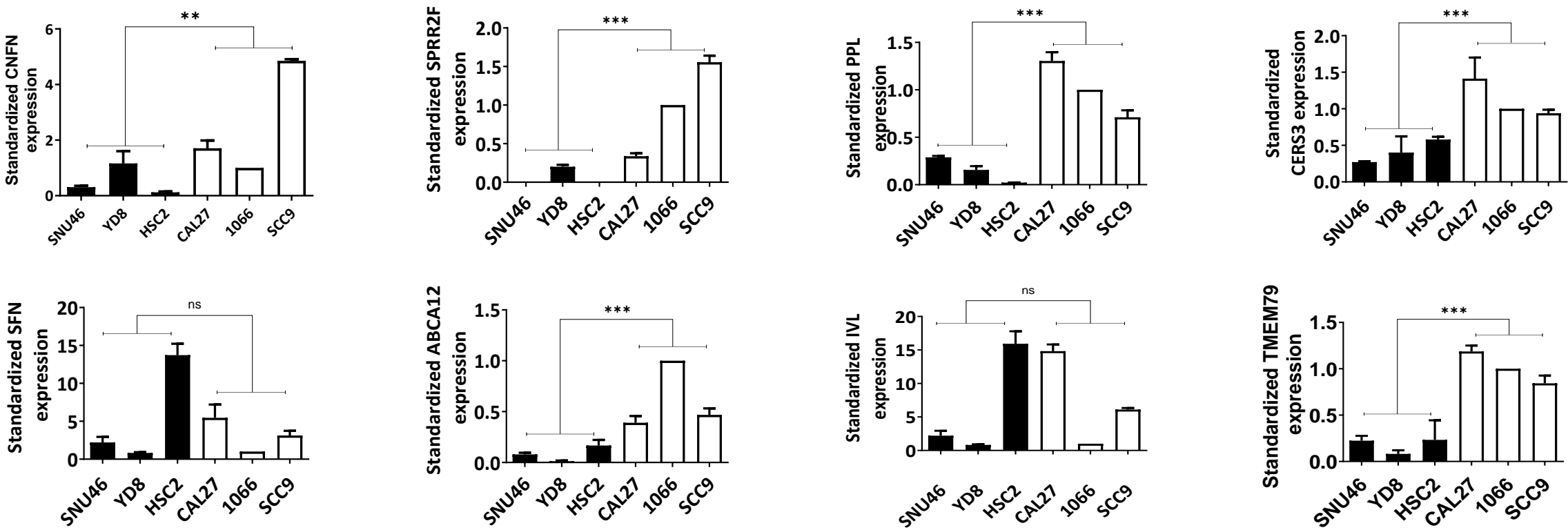

Supplement: Supplementary file 5 — Supplementary Material 5: Figure 5. Expression profiles of keratinization-related genes in HNSCC cell lines.Expression levels of eight keratinization-related genes from CCLE data in six HNSCC cell lines. Statistical significance was determined unpaired Student’s t-test. *p < 0.05; **p < 0.01; ***p < 0.001 Expression levels of eight keratinization-related genes in six HNSCC cell lines were analyzed by RT-PCR [file 11658_2025_855_MOESM5_ESM.pdf]

Supplementary Figure 6

A

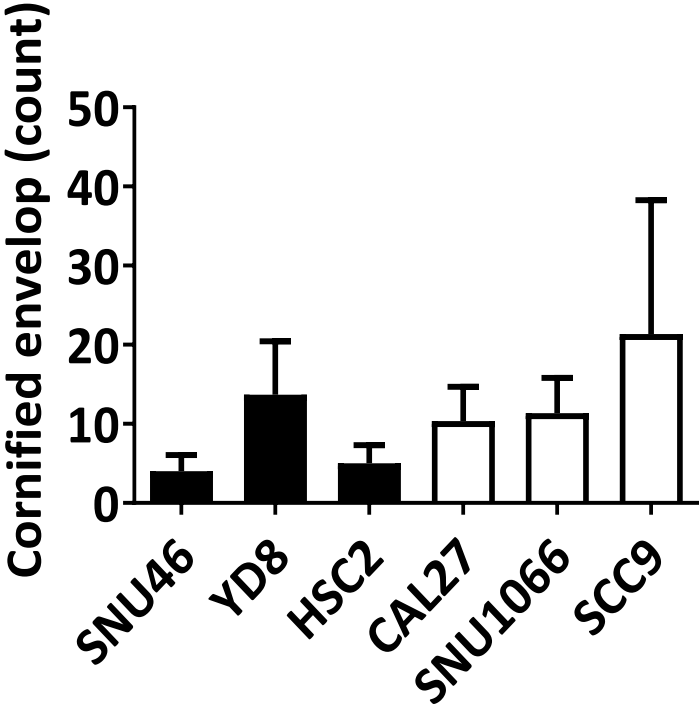

B

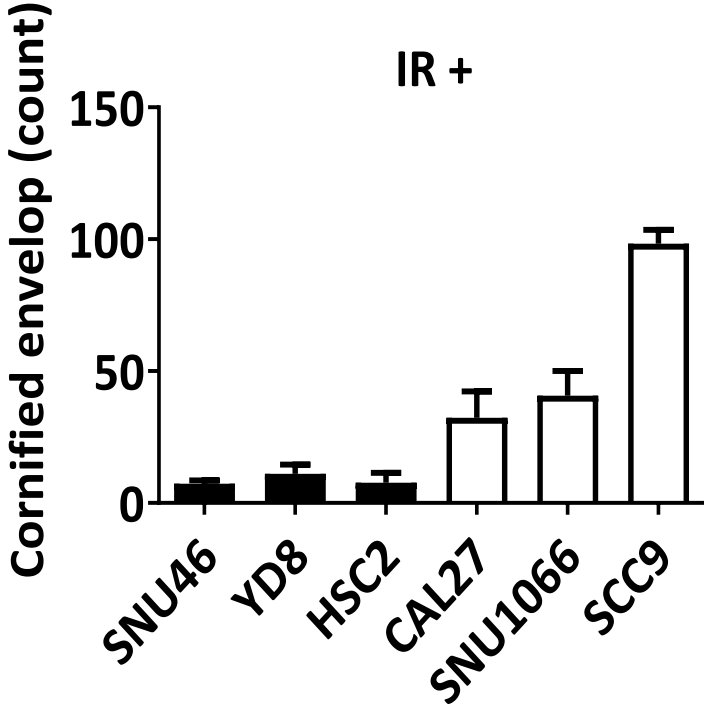

Supplement: Supplementary file 6 — Supplementary Material 6: Figure 6. Assessment of cornified envelope formation in HNSCC cell lines.Cornified envelope assay showing cornified envelope formation in six cell lines without radiation exposure. Cornified envelopes were counted under a microscope. The x-axis represents HNSCC cell lines, and the y-axis represents the number of cornified envelopes. Error bars represent SD.Cornified envelope assay showing cornified envelope formation in six cell lines after radiation exposure. Cornified envelopes were counted under a microscope. The x-axis represents HNSCC cell lines, and the y-axis represents the number of cornified envelopes. Error bars represent SD. [file 11658_2025_855_MOESM6_ESM.pdf]

Supplementary Figure 7

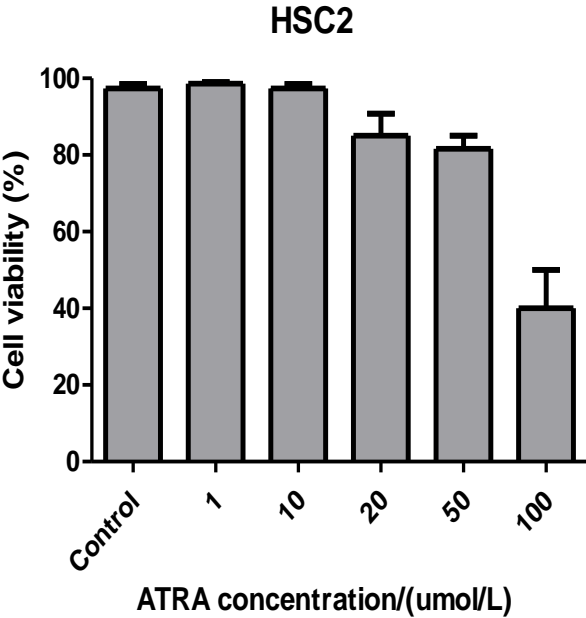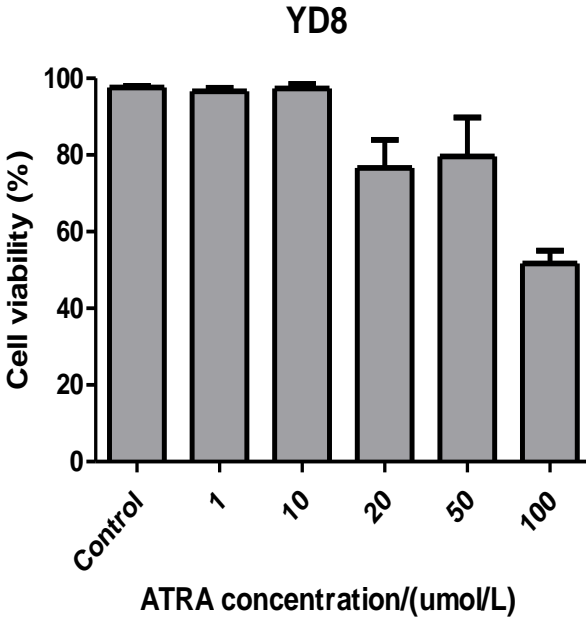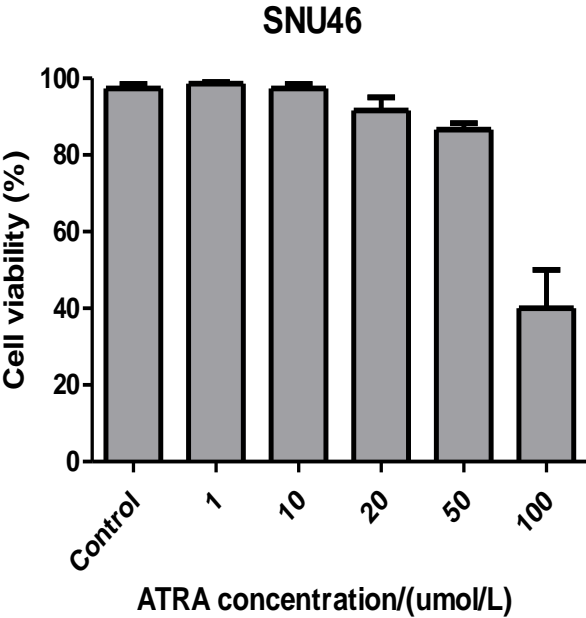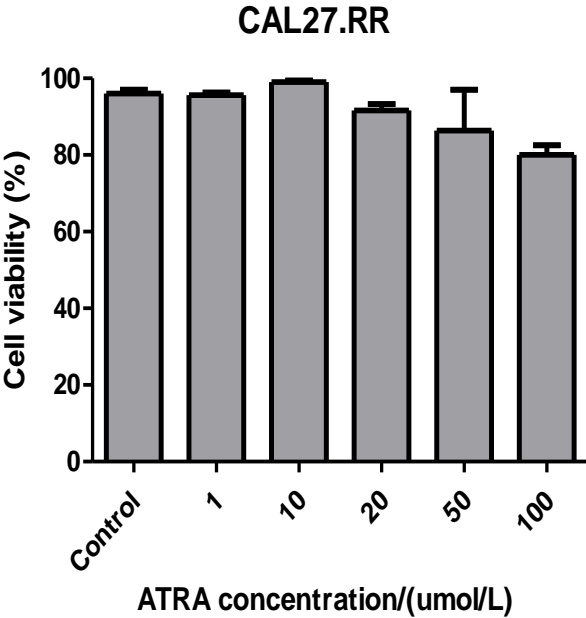

Supplement: Supplementary file 7 — Supplementary Material 7: Figure 7. Viability of ATRA in HNSCC. MTT assay results demonstrating the effect of ATRA on cell viability in HNSCC cells. Cells were treated with various concentrations of ATRA for 24 h. Values represent the mean ± SD of three independent experiments. Error bars represent SD. Cell viability was measured by reading absorbance at 570 nm using a microplate reader. [file 11658_2025_855_MOESM7_ESM.pdf]

Supplementary Figure 8

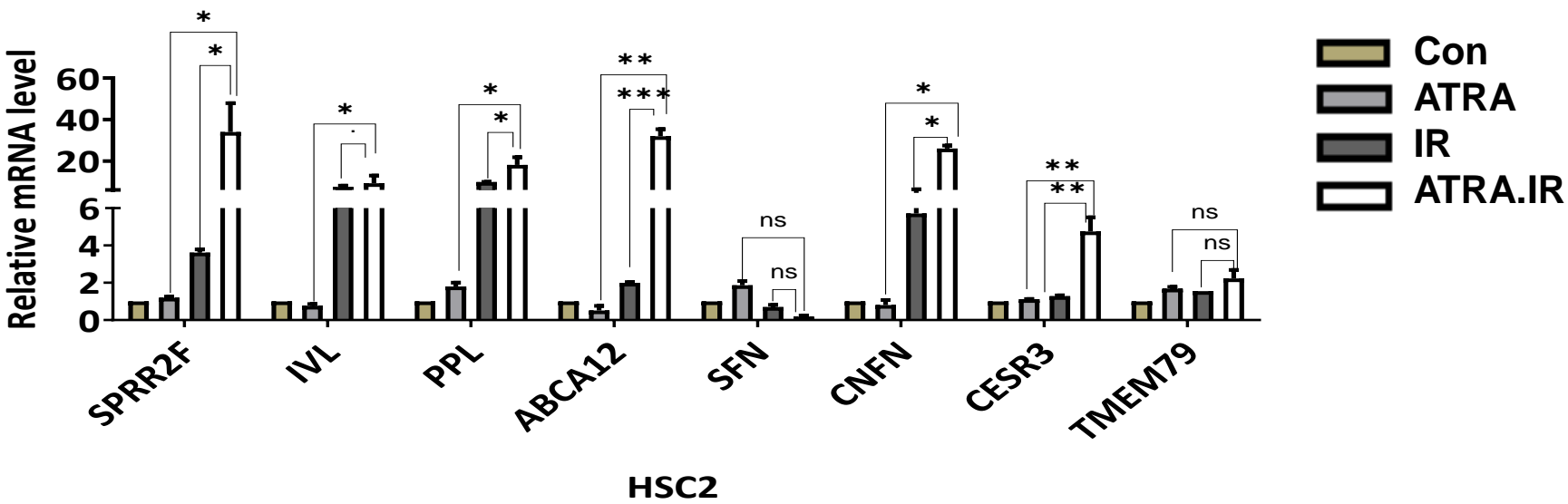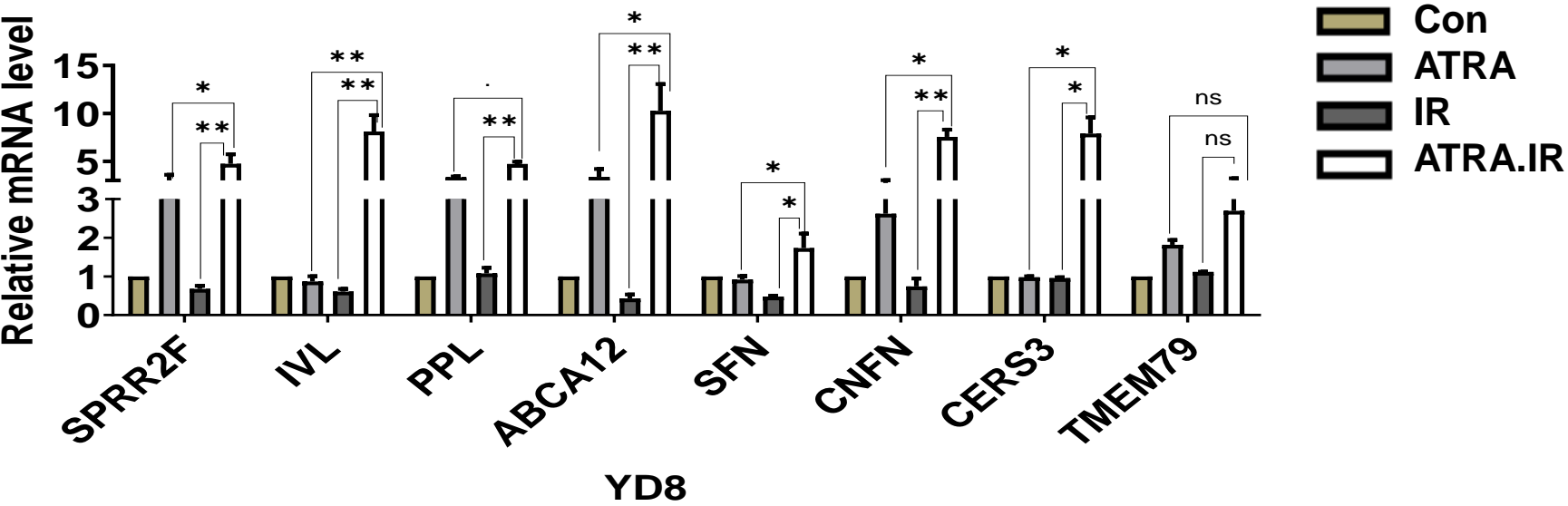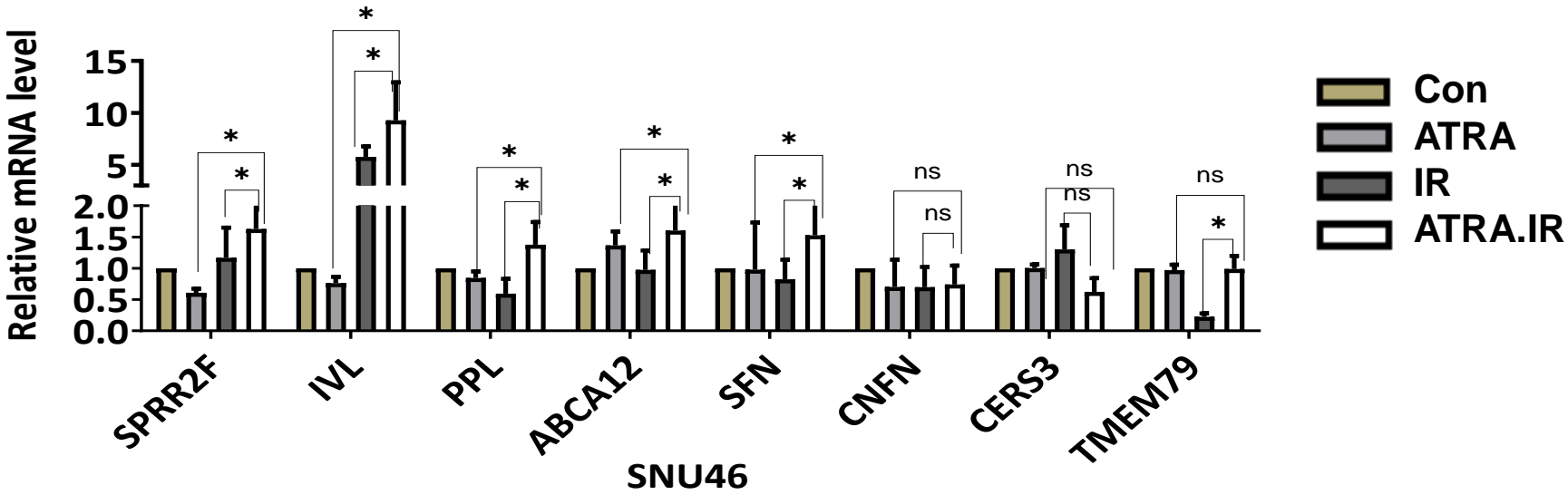

Supplement: Supplementary file 8 — Supplementary Material 8: Figure 8. Relative mRNA expression levels of keratinization-related genes in KRGSlow cell lines. The x-axis indicates the analyzed genes, and the y-axis shows the relative mRNA levels normalized to control. Bar colors represent treatment groups as follows: gold for the untreated control, light gray for ATRA treatment, dark gray for irradiation, and black for the combined ATRA and irradiation treatment. Cells were treated with ATRA for 24 h, and statistically significant were determined unpaired Student’s t-test. *p < 0.05; **p < 0.01; *p < 0.001. Data are presented as mean ± SD. [file 11658_2025_855_MOESM8_ESM.pdf]

Supplementary Figure 9

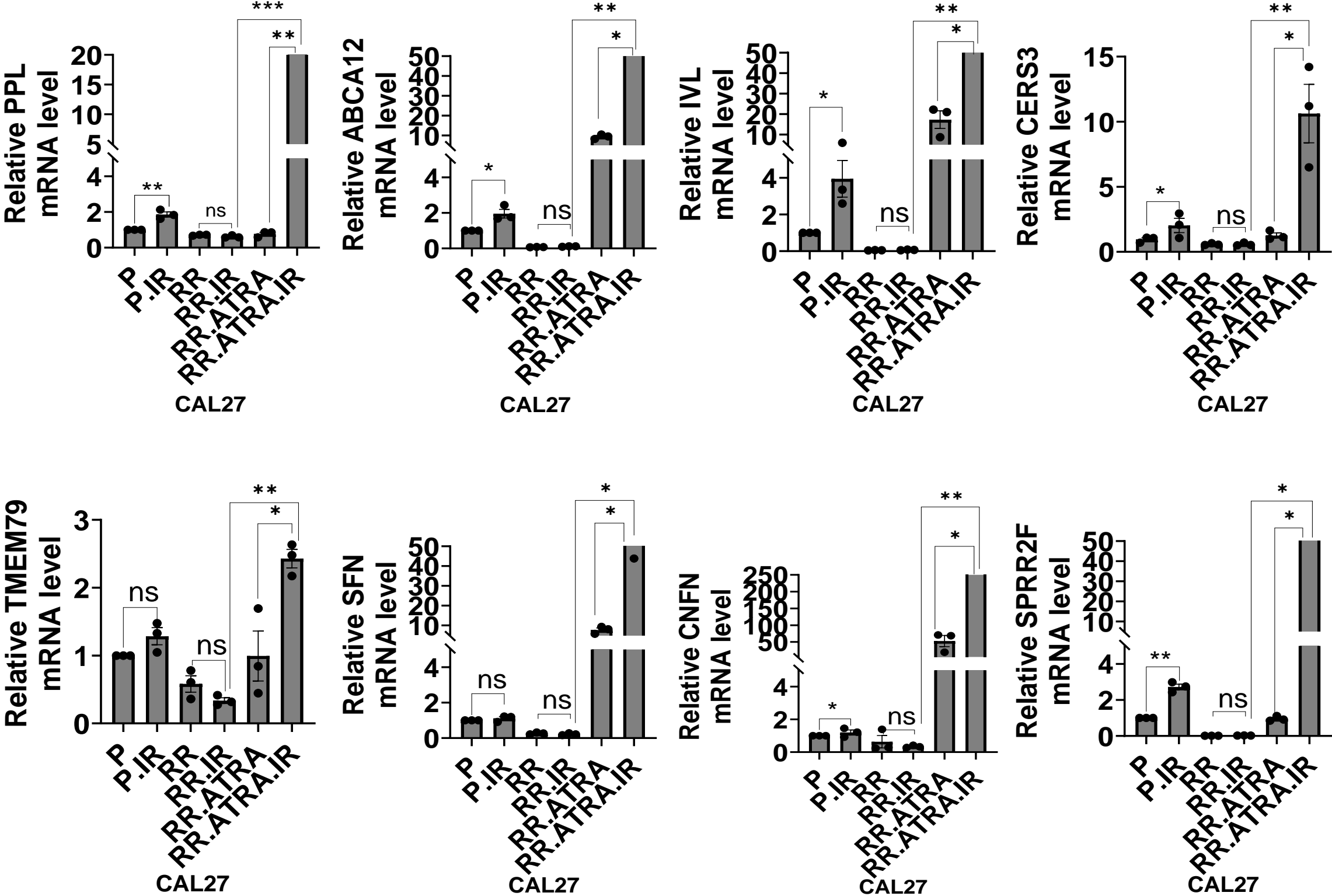

Supplement: Supplementary file 9 — Supplementary Material 9: Figure 9. Relative mRNA expression levels of keratinization-related genes in CAL27-P and CAL27-RR cells. The x-axis represents the experimental groups: P, P.IR, RR, RR.IR, RR.ATRA, and RR.ATRA.IR. The y-axis shows the relative mRNA expression levels normalized to the untreated control. Cells were treated with ATRA for 24 h. Data are presented as mean ± SD, and statistically significant were determined unpaired Student’s t-test. *p < 0.05; **p < 0.01; *p < 0.001. [file 11658_2025_855_MOESM9_ESM.pdf]

**Supplementary Figure 10**

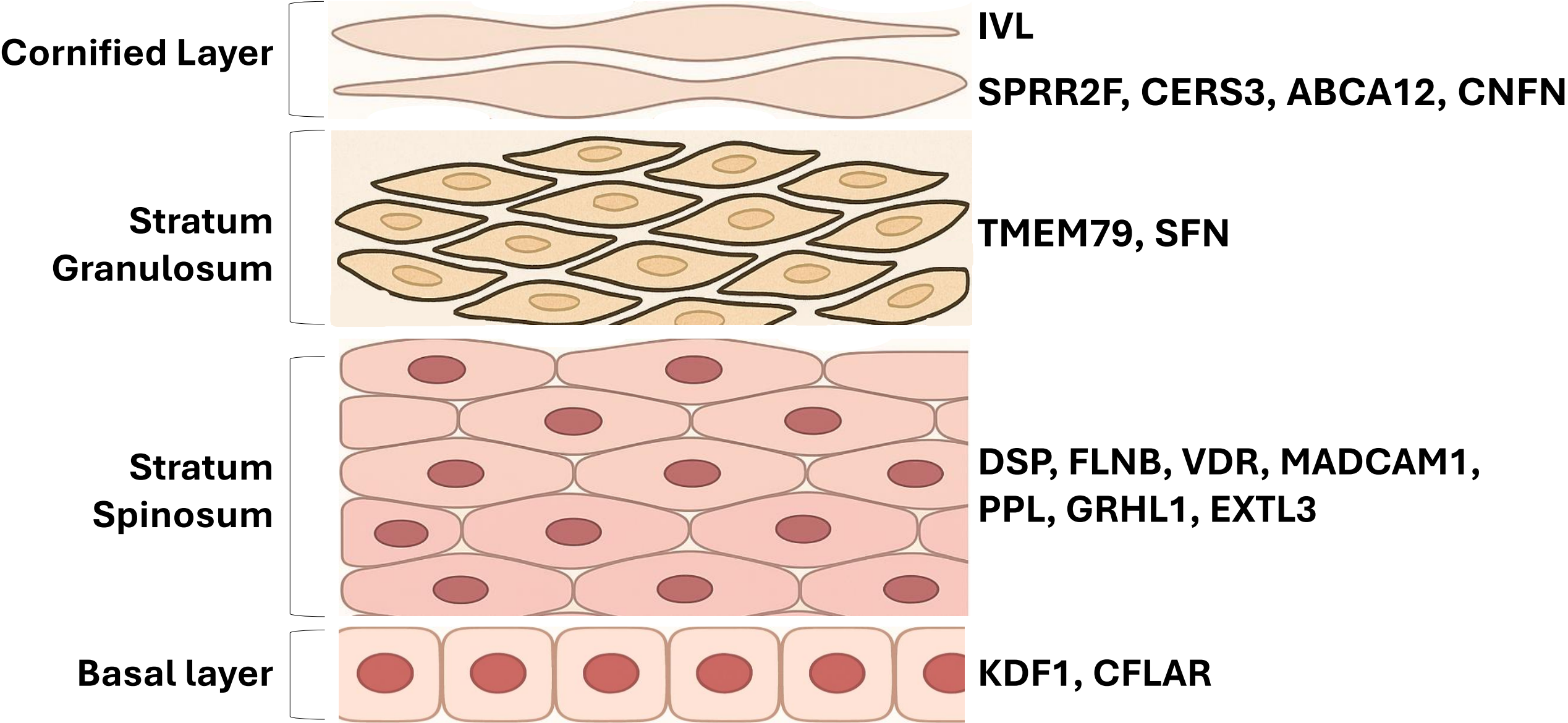

Supplement: Supplementary file 10 — Supplementary Material 10: Figure 10. Schematic visualization of keratinization progressing to cornification in relation to KRGS genes [file 11658_2025_855_MOESM10_ESM.pdf]

Supplementary Figure 11

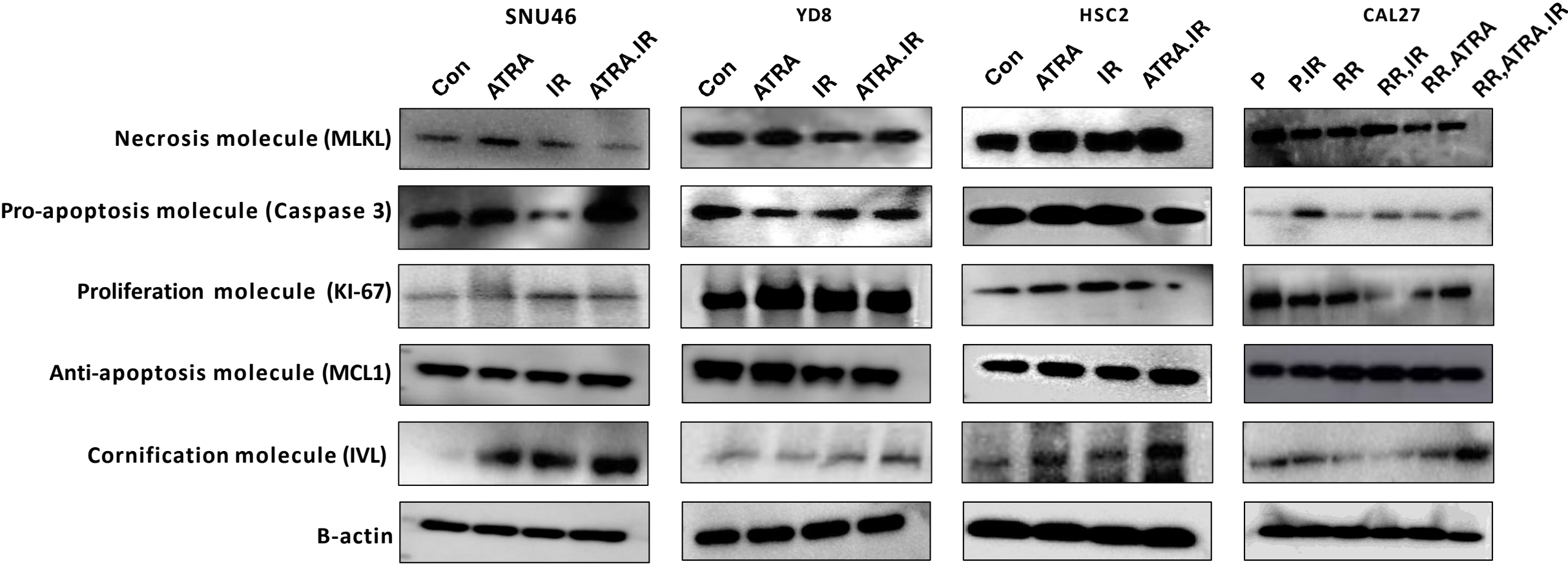

Supplement: Supplementary file 11 — Supplementary Material 11: Figure 11. Cell death mechanism induced by the combination of keratinization activation and IR through IVL expression in HNSCC. Protein levels in HNSCC cell lines were determined by Western blot. β-Actin was used as an internal loading control. The experiment was performed at least three times [file 11658_2025_855_MOESM11_ESM.pdf]

Supplementary Figure 13

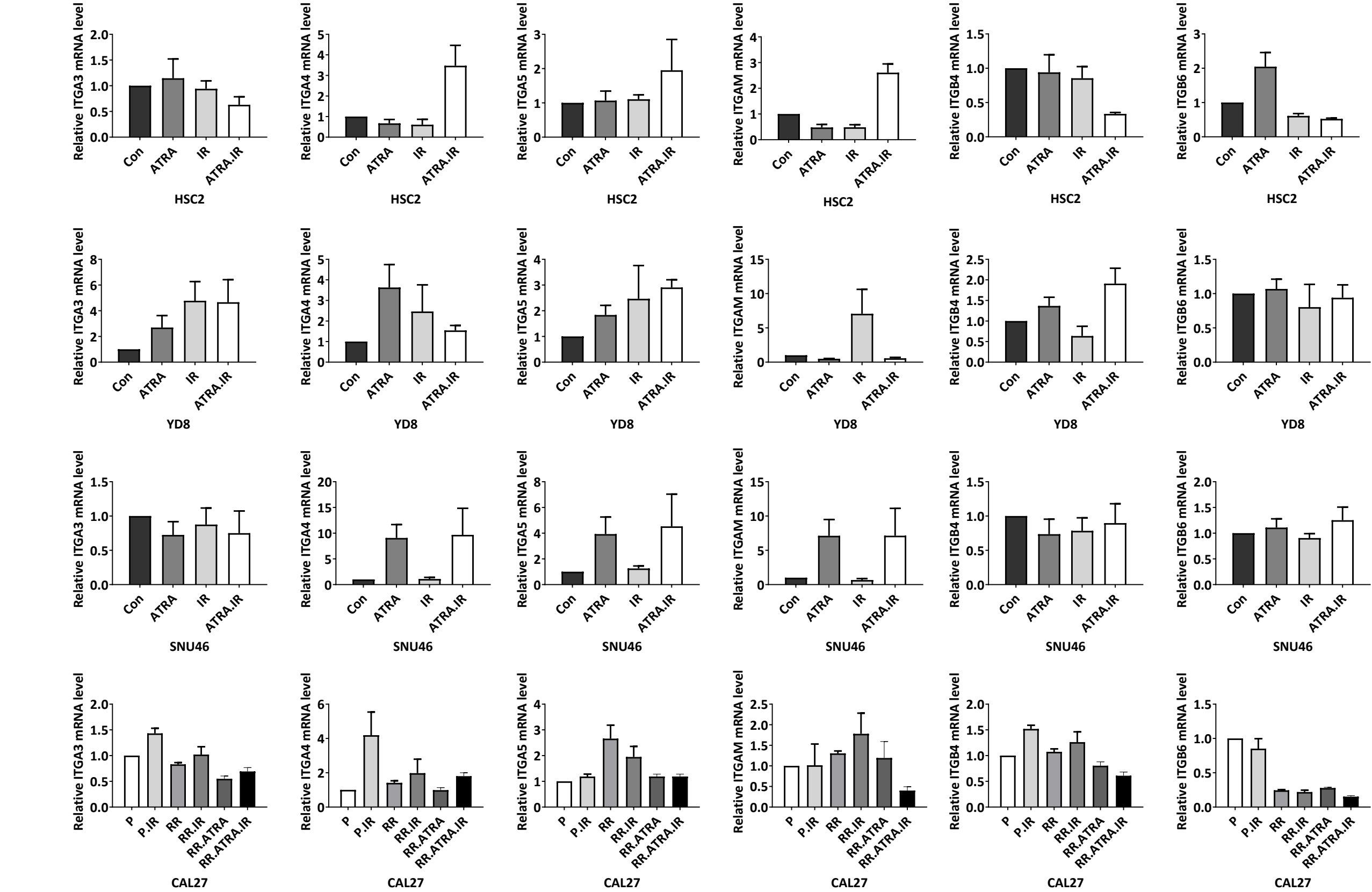

Supplement: Supplementary file 13 — Supplementary Material 13: Figure 13. Expression changes of integrin genes upon ATRA treatment and IR exposure. mRNA expression levels of six integrin genesin KRGSlow, CAL27-P, and CAL27-RR HNSCC cell lines were analyzed by RT-PCR. Experiments were performed at least three times. Cells were treated with ATRA for 24 h. The x-axis represents the treatment groups: Con, ATRA, IR, and ATRA.IR. The y-axis shows relative mRNA expression levels normalized to the control. Data are presented as mean ± SD. [file 11658_2025_855_MOESM13_ESM.pdf]

Supplementary Figure 14

A

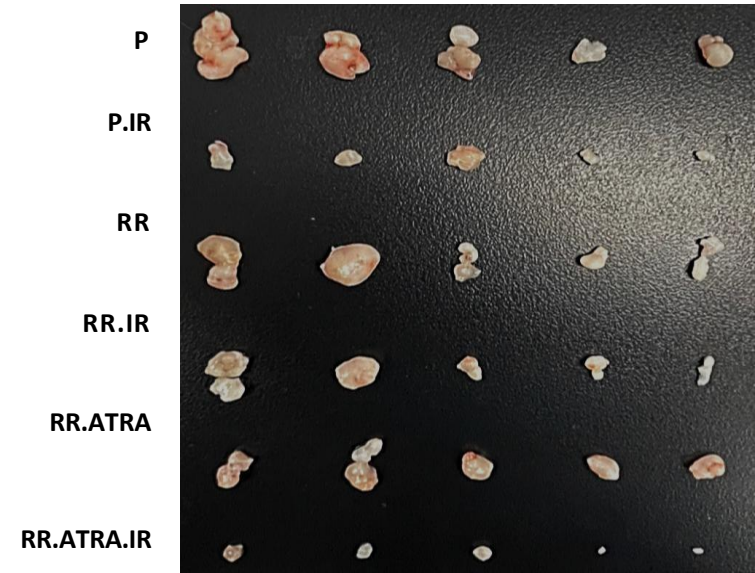

B

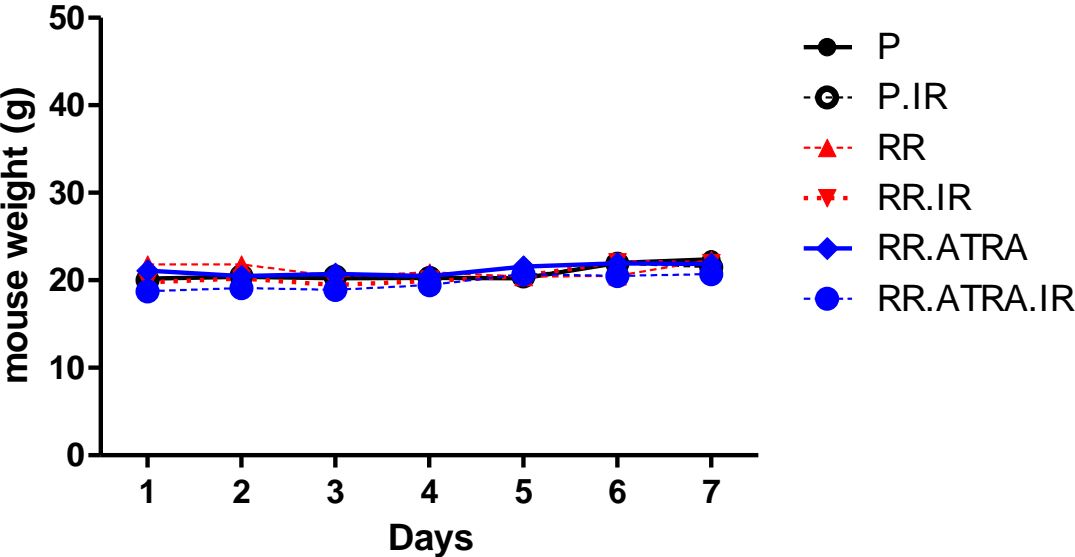

Supplement: Supplementary file 14 — Supplementary Material 14: Figure 14. Impact of treatment on tumor growth and systemic effects in mouse models.Tumor-bearing mice were categorized into two groups based on tumor type: CAL27-P and CAL27-RR. Mice with CAL27-P tumors received either irradiationor no treatment, mice with CAL27-RR tumors were treated with ATRA, IR, a combination of two agents, or left untreated as controls. Representative photographs of dissected tumors from each experimental group are presented. Mouse body weights were recorded at consistent intervals throughout the experiment to monitor any treatment-related systemic toxicity or adverse effects. Mouse body weights were measured twice a week [file 11658_2025_855_MOESM14_ESM.pdf]

Supplementary Figure 15

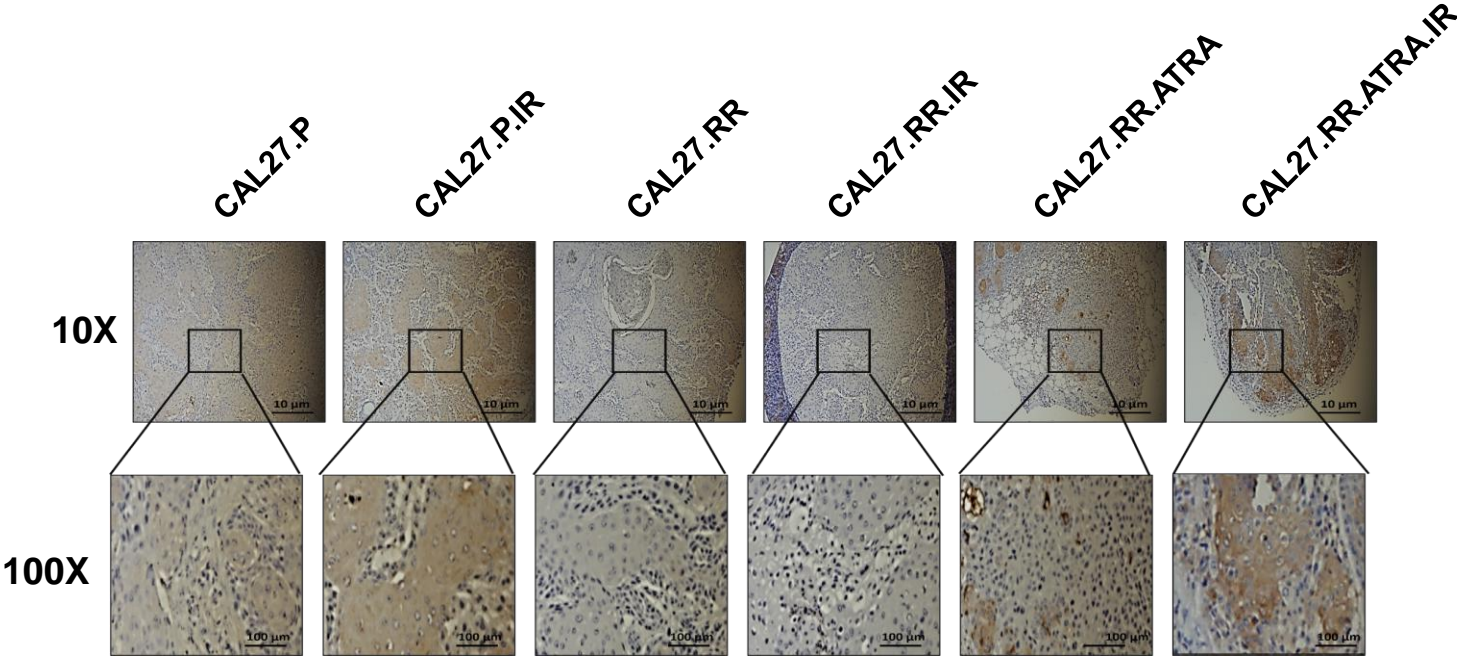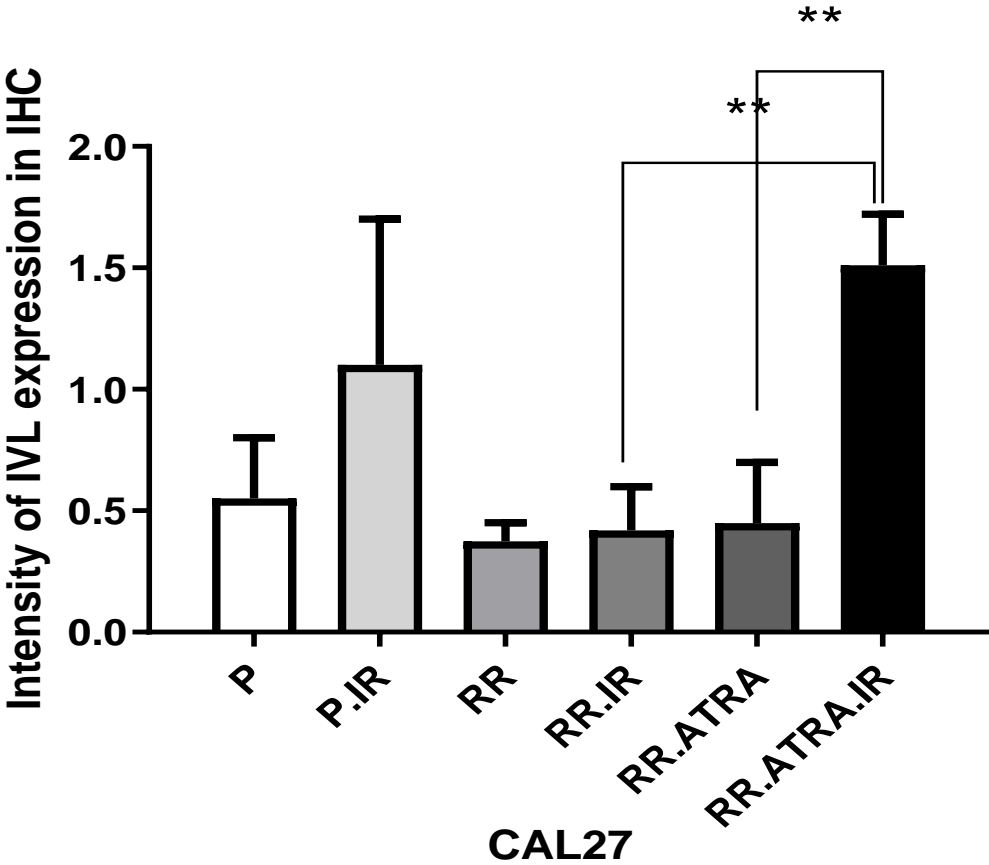

Supplement: Supplementary file 15 — Supplementary Material 15: Figure 15. IHC analysis of IVL expression in xenograft tumor tissues.Representative IHC staining images of xenograft tumor tissues from the following mouse groups: CAL27.P, CAL27.P IR, CAL27.RR, CAL27.RR ATRA, CAL27.RR IR, and CAL27.RR ATRA.IREach column shows paired low-magnificationand high-magnificationviews of the same region.These images were analyzed to assess the intensity of IVL expression levels in response to the different treatment conditions. Image Jwas performed to measure intensity of IVL. Statistical significance of differences in IVL expression was determined using unpaired Student’s t-test. *p < 0.05; **p < 0.01; *p < 0.001. [file 11658_2025_855_MOESM15_ESM.pdf]

Supplementary Figure 16

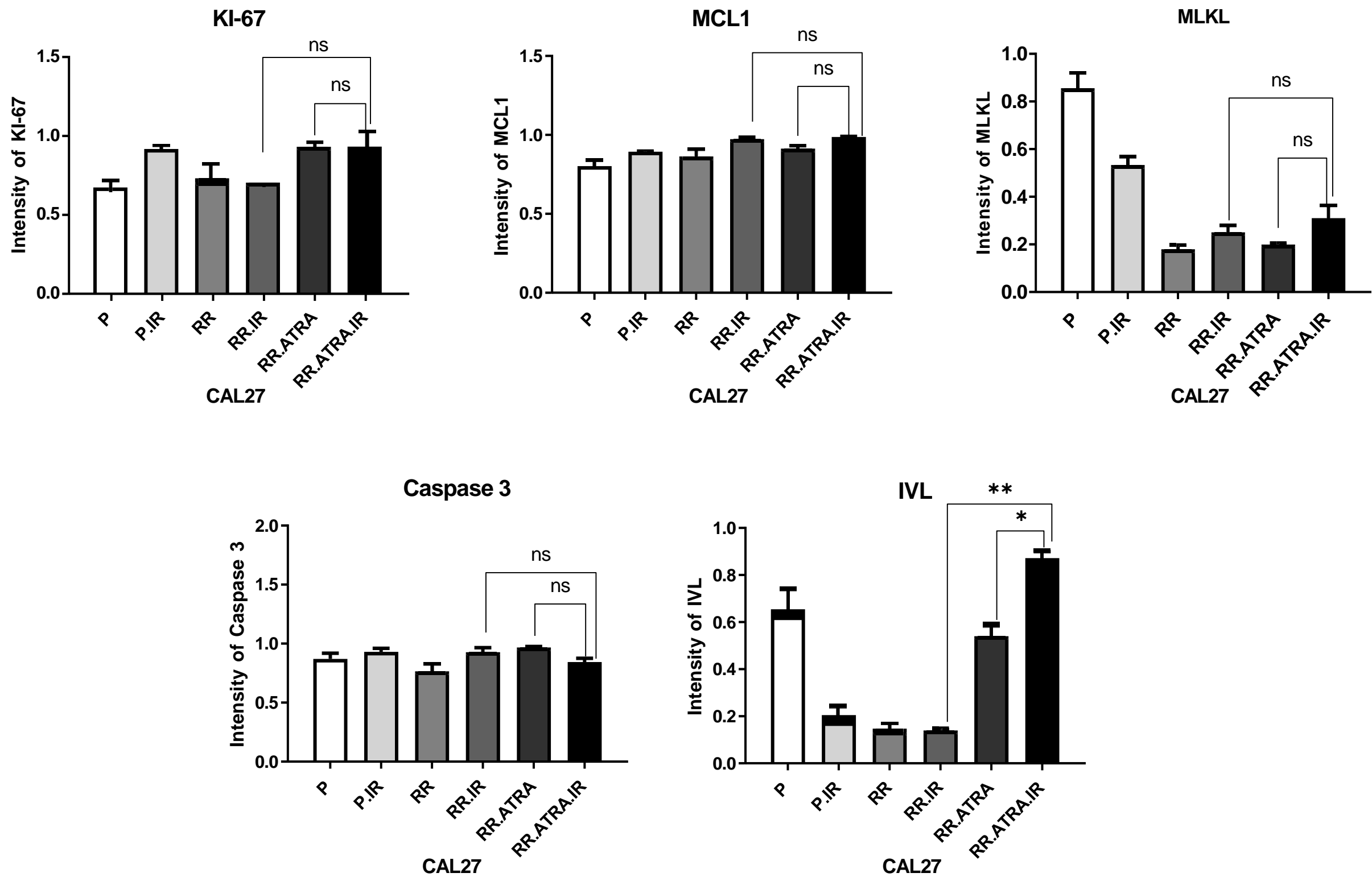

Supplement: Supplementary file 16 — Supplementary Material 16: Figure 16. Quantification of protein from western blot. Protein expression levels in xenograft tissues from the following mouse groups were quantified: CAL27.P, CAL27.P IR, CAL27.RR, CAL27.RR ATRA, CAL27.RR IR, and CAL27.RR ATRA.IR. The x-axis represents the different treatment groups, and the y-axis represents the intensity of protein quantification. Statistical significance was determined using an unpaired Student’s t-test. *p < 0.05; **p < 0.01; ***p < 0.001. [file 11658_2025_855_MOESM16_ESM.pdf]

Supplementary Figure 17

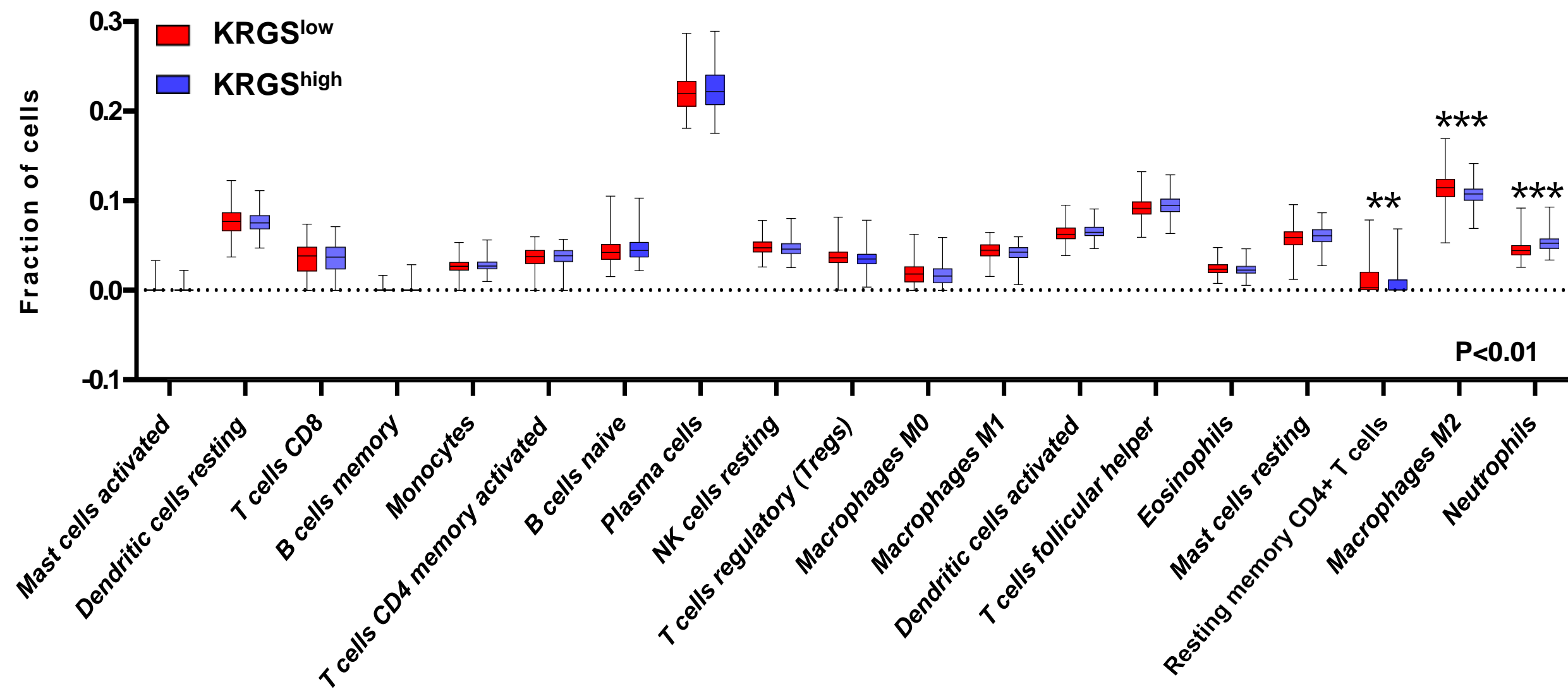

Supplement: Supplementary file 17 — Supplementary Material 17: Figure 17. Estimation of immune cell proportions in KRGS subgroups. Proportions of 19 immune cell types, estimated using CIBERSORT were analyzed in HNSCC from the TCGA database, comparing KRGSlow and KRGShigh of the KRGS. Statistical significance was determined using an unpaired Student’s t-test. *p < 0.05; **p < 0.01; ***p < 0.001. [file 11658_2025_855_MOESM17_ESM.pdf]
