# Supplementary material for: Keratinization-related gene signature predicting survival and response to radiation in patients with HPV-negative head and neck squamous cell carcinoma via regulation of cornification and integrin signaling
Source: Cell Mol Biol Lett. 2026 Jan 29;31:26. doi: 10.1186/s11658-025-00855-y (PMC12924225; doi:10.1186/s11658-025-00855-y)
Supplement: Supplementary file 12 — Supplementary Material 12: Figure 12. Differential expression of integrin genes between CAL27-P and CAL27-RR cells.Volcano plot of differentially expressed integrin genes between CAL27-P and CAL27-RR in RNA sequencing data. The y-axis represents the p-value, and the x-axis represents the fold change.The six significantly differentially expressed integrin genes between CAL27-P and CAL27-RR [file 11658_2025_855_MOESM12_ESM.pdf]

Supplementary Figure 12

A

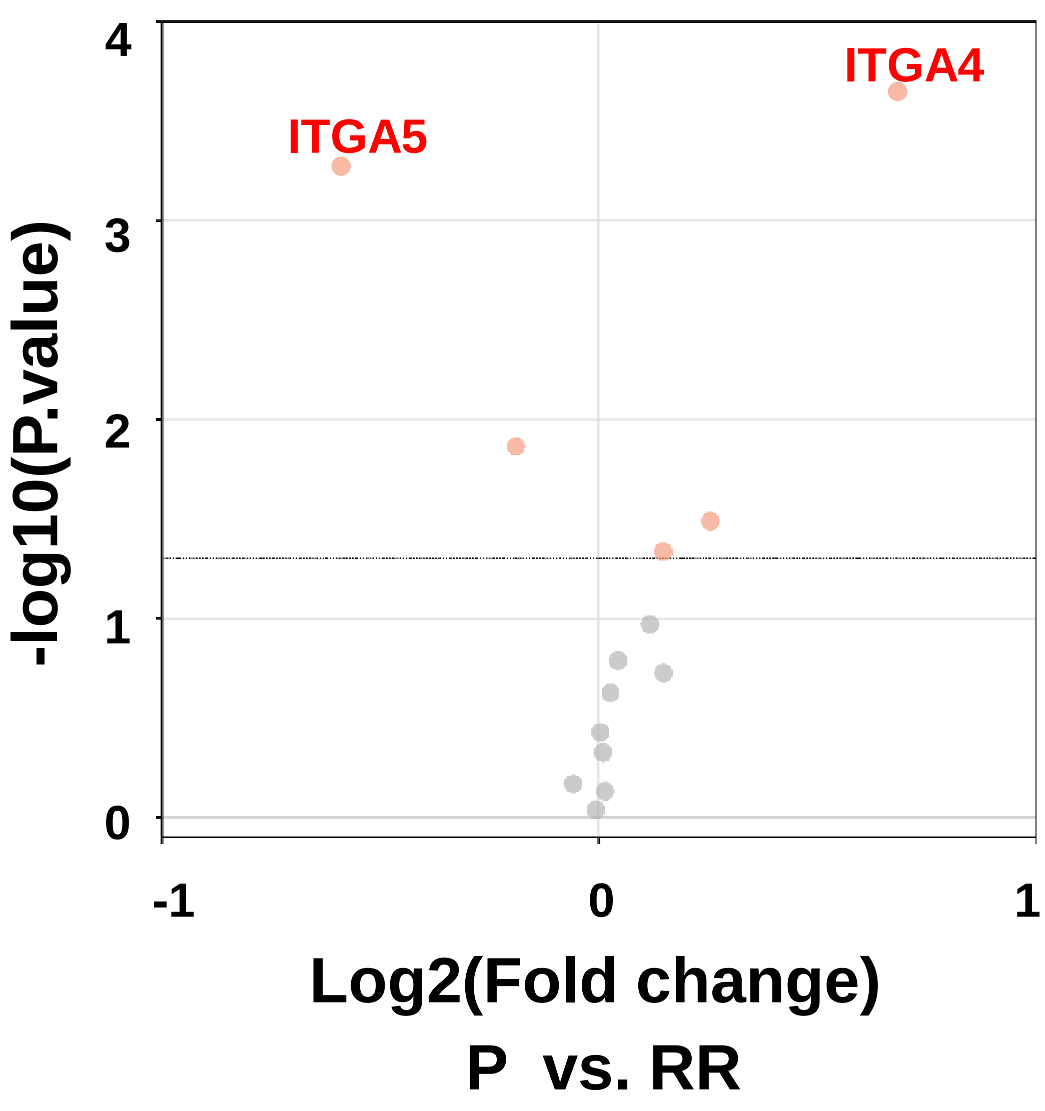

B

Gene_Symbol	FC	P.value
**ITGA4**	**1.517848**	**0.000133**
**ITGA5**	**-1.12337**	**0.000455**
**ITGB4**	**0.838916**	**0.001346**
**ITGB6**	**-0.66267**	**0.011661**
**ITGAM**	**-0.3788**	**0.013662**
**ITGA1**	**0.511129**	**0.032415**
**ITGA3**	**0.295607**	**0.046266**
ITGB7	0.126357	0.066232
ITGB8	0.547897	0.08769
ITGA6	0.234786	0.106984
ITGAV	0.296923	0.187901
ITGAL	0.05373	0.237008
ITGB1	0.148759	0.287757
ITGAX	0.006813	0.373901
ITGB3	-0.00671	0.373901
ITGB5	-0.20298	0.374947
ITGB2	-0.10381	0.495124
ITGA2	-0.11656	0.679928
ITGA2B	0.02891	0.741585
ITGAE	-0.01319	0.916255
