# Supplementary material for: Keratinization-related gene signature predicting survival and response to radiation in patients with HPV-negative head and neck squamous cell carcinoma via regulation of cornification and integrin signaling
Source: Cell Mol Biol Lett. 2026 Jan 29;31:26. doi: 10.1186/s11658-025-00855-y (PMC12924225; doi:10.1186/s11658-025-00855-y)
Supplement: Supplementary file 18 — Supplementary Material 18. [file 11658_2025_855_MOESM18_ESM.docx]

**Table S1. Clinical and pathological features of HNSCC in 5 cohorts.**

|  | **TCGA** | **FHCRC** | **Leipzig** | **MDACC** | **KHU** |
| --- | --- | --- | --- | --- | --- |
| Number of patients | 452 | 97 | 196 | 74 | 47 |
| Gender |  |  |  |  |  |
| Male | 322 (71.2%) | 66 (68.0%) | 167 (85.2%) | 58 (78.4%) | 40 (85.1%) |
| Female | 129 (28.5%) | 31 (32.0%) | 29 (14.7%) | 16 (21.6%) | 7 (14.8%) |
| Age  (mean ± SD) | 61.5±12.12 | NA | 60.1±10.8 | 58.1±13.6 | 64.1±11.5 |
| Anatomic site |  |  |  |  |  |
| Oral cavity | 300 (66.3%) | 86 (88.7%) | 70 (35.7%) | 71 (95.9%) | 37 (78.7%) |
| Oropharynx | 31 (6.8%) | 11 (11.3%) | 47 (24.0%) | 3 (4.1%) | 0 (0.0%) |
| Larynx | 112 (24.8%) | 0 | 45 (23.0%) | 0 | 7 (14.9%) |
| Hypopharynx | 8 (1.8%) | 0 | 30 (15.3%) | 0 | 2 (4.3%) |
| Others |  |  | 4 (2.0%) | 0 |  |
| Primary tumor |  |  |  |  |  |
| T1 | 41 (9.1%) | NA | 25 (12.7%) | 3 (4.1%) | 6 (12.7%) |
| T2 | 115 (25.4%) | NA | 53 (27.0%) | 27 (36.5%) | 18 (38.3%) |
| T3 | 93 (20.6%) | NA | 46 (23.4%) | 28 (37.8%) | 6 (12.7%) |
| T4 | 163 (36.1%) | NA | 72 (36.7%) | 16 (21.6%) | 24 (51.1%) |
| Regional lymph node |  |  |  |  |  |
| N0 | 162 (35.8%) | NA | 81 (40.1%) | NA | 19 (40.4%) |
| N1 | 60 (13.2%) | NA | 24 (12.2%) | NA | 6 (12.7%) |
| N2 | 148 (32.7%) | NA | 83 (42.3%) | NA | 18 (38.3%) |
| N3 | 8 (1.8%) | NA | 8 (4.1%) | NA | 1 (2.1%) |
| Stage |  |  |  |  |  |
| I | 18 (4.0%) | 30 (30.9%) | 16 (8.2%) | 3 (4.1%) | 2 (4.3%) |
| II | 87 (19.2%) | 22 (11.3%) | 31 (15.8%) | 16 (21.6%) | 7 (14.9%) |
| III | 97 (21.4%) | 26 (15.5%) | 28 (14.3%) | 15 (20.3%) | 5 (10.6%) |
| IV | 235 (52.0%) | 52 (42.3%) | 121 (61.7%) | 40 (54.1%) | 16 (34.0%) |
| Tobacco use |  |  |  |  |  |
| Never | 11 (2.4%) | NA | 27 (13.7%) | 15 (20.3%) | 15 (31.9%) |
| Yes | 440 (97.3%) | NA | 169 (86.2%) | 59 (79.7%) | 31 (65.0%) |
| Alcohol use |  |  |  |  |  |
| Never | 148 (32.7%) | NA | 21 (10.7%) | NA | NA |
| Yes | 293 (68.4%) | NA | 175 (89.2%) | NA | NA |
| KRGS |  |  |  |  |  |
| KRGS^low^ | 239 (52.8%) | 44 (45.2%) | 82 (41.8%) | 27 (36.0%) | 27 (57.4%) |
| KRGS^high^ | 212 (46.9%) | 53 (54.6%) | 114 (58.2%) | 41 (54.7%) | 20 (42.5%) |
