# Supplementary material for: Keratinization-related gene signature predicting survival and response to radiation in patients with HPV-negative head and neck squamous cell carcinoma via regulation of cornification and integrin signaling
Source: Cell Mol Biol Lett. 2026 Jan 29;31:26. doi: 10.1186/s11658-025-00855-y (PMC12924225; doi:10.1186/s11658-025-00855-y)
Supplement: Supplementary file 19 — Supplementary Material 19. [file 11658_2025_855_MOESM19_ESM.docx]

**Table S2. Primer sequences for RT-PCR**

| **Gene** | **Forward primer sequence(5’-3’)** | **Reverse primer sequence(3’-5’)** |
| --- | --- | --- |
| CNFN | ATC GCG ATC CTG CAG CTA TG | CAA AAG TGC CGC ACA GAC AG |
| SPRR2F | TAA GCA CCG ATC TGC CTT GG | AGG AGG ACA TTT CTG CTG GC |
| IVL | GGG ACT GCC TGA GCA AGA AT | GGA GCT CCA ACA GTT GCT CT |
| SFN  ABCA12  PPL  TMEM79  CERS3  ITGA1  ITGA3  ITGA4  ITGA5  ITGAM  ITGB4  ITGB6 | TGG AGG GTG CTG TCC AGT AT  AGA ACA CCA ACC CAT CTG CC  CAG ACC CGG AGC ATC TCT AAC  GGA GAT CCA CCG GCG ATA TG  GTG CTC GCA CAG ATG GTG T  GGGAAGCTGCCAGTGAGATT  GTACACGATGCAGGTAGGCA  AGCTGGGTAGCCCTAATGGA  CGGGGGCTTCAACTTAGACG  CTCCTTCCAGGTTCTGGCTC  CTGCAGCCCCATCTCCTAGC  ATCGGTCTGCACAGCAAGAA | CTT CAG GTA GAA GAC CCG GC  TCC GTA CCG TGT GTA GGT CT  GTC ACT CTG CAT CTT GGC CT  TGT CAG ACC CAA GCC GCT AC  TCC AAC GTT CCA ACC AGC TT  GCAGCAGCGTAGAACAACAG  ATTGTTCAGGTCTGCCAGGG  TCACCCCAATTCTGCTCACT  ATTCAATGGGGGTGCACTGT  CAGGAAGCAGAGCCCTTTCA  AAGCTGCTCTCCATGACCAC  GGGTATCACACCTTTCGCCA |
